# Effects of tocotrienols supplementation on markers of inflammation and oxidative stress: A systematic review and meta-analysis of randomized controlled trials

**DOI:** 10.1371/journal.pone.0255205

**Published:** 2021-07-23

**Authors:** Ban-Hock Khor, Hui-Ci Tiong, Shing Cheng Tan, Sok Kuan Wong, Kok-Yong Chin, Tilakavati Karupaiah, Soelaiman Ima-Nirwana, Abdul Halim Abdul Gafor

**Affiliations:** 1 Faculty of Medicine, Department of Medicine, Universiti Kebangsaan Malaysia, Cheras, Kuala Lumpur, Malaysia; 2 Faculty of Food Science and Nutrition, Universiti Malaysia Sabah, Kota Kinabalu, Sabah, Malaysia; 3 UKM Medical Molecular Biology Institute, Universiti Kebangsaan Malaysia, Cheras, Kuala Lumpur, Malaysia; 4 Faculty of Medicine, Department of Pharmacology, Universiti Kebangsaan Malaysia, Cheras, Kuala Lumpur, Malaysia; 5 Faculty of Health and Medical Sciences, School of Biosciences, Taylor’s University, Subang Jaya, Selangor, Malaysia; University College London, UNITED KINGDOM

## Abstract

Studies investigating the effects of tocotrienols on inflammation and oxidative stress have yielded inconsistent results. This systematic review and meta-analysis aimed to evaluate the effects of tocotrienols supplementation on inflammatory and oxidative stress biomarkers. We searched PubMed, Scopus, and Cochrane Central Register of Controlled Trials from inception until 13 July 2020 to identify randomized controlled trials supplementing tocotrienols and reporting circulating inflammatory or oxidative stress outcomes. Weighted mean difference (WMD) and corresponding 95% confidence interval (CI) were determined by pooling eligible studies. Nineteen studies were included for qualitative analysis, and 13 studies were included for the meta-analyses. A significant reduction in C-reactive protein levels (WMD: −0.52 mg/L, 95% CI: −0.73, −0.32, *p* < 0.001) following tocotrienols supplementation was observed, but this finding was attributed to a single study using δ-tocotrienols, not mixed tocotrienols. There were no effects on interleukin-6 (WMD: 0.03 pg/mL, 95% CI: −1.51, 1.58, *p* = 0.966), tumor necrosis factor-alpha (WMD: −0.28 pg/mL, 95% CI: −1.24, 0.68, *p* = 0.571), and malondialdehyde (WMD: −0.42 μmol/L, 95% CI: −1.05, 0.21, *p* = 0.189). A subgroup analysis suggested that tocotrienols at 400 mg/day might reduce malondialdehyde levels (WMD: −0.90 μmol/L, 95% CI: −1.20, −0.59, *p* < 0.001). Future well-designed studies are warranted to confirm the effects of tocotrienols on inflammatory and oxidative stress biomarkers, particularly on different types and dosages of supplementation. PROSPERO registration number: CRD42020198241.

## Introduction

Inflammation is an essential immune response for immunosurveillance and host defense. While acute inflammation is a beneficial and self-limiting process to eliminate toxic agents or promote the repair of damaged tissue, chronic inflammation is a pathological state associated with a homeostatic imbalance of physiological systems not directly triggered by infection or injury [1,2]. On the other hand, oxidative stress is a phenomenon that denotes an imbalance between the production and accumulation of free radicals or oxidants and the antioxidant system to counteract these reactive products [3]. Chronic inflammation and oxidative stress are two pathophysiological conditions that coexist because they can induce each other mutually [4]. Chronic inflammation and oxidative stress disrupt normal cellular physiology. They have been implicated in the development and progression of various metabolic or chronic diseases such as metabolic syndrome and diabetes, cardiovascular disease, cancers, neurodegenerative disorders, chronic kidney disease, liver disease, and rheumatoid arthritis [3,5,6].

Vitamin E, also known as α-tocopherol, is a lipid-soluble vitamin with potent antioxidant and anti-inflammatory properties. Tocopherols possess a saturated wide chain while tocotrienols differ from tocopherols by possessing an isoprenoid side chain with three double bonds. [7,8]. Tocotrienols are further divided into four distinct isomers: alpha (α), beta (β), gamma (γ), and delta (δ). Till now, most research has focused primarily onα-tocopherol. However, there is a growing interest in exploring the potential role of tocotrienols in preventing and treating chronic diseases [9]. Tocotrienols are reported to possess more superior antioxidant properties than α-tocopherol due to the presence of the unsaturated side chain, which allows more efficient incorporation into tissues with saturated fatty layers such as the brain and liver [10]. Beyond the antioxidant capacity, tocotrienols are also shown to modulate inflammatory responses via the regulation of gene expression of pro-inflammatory cytokines [11]. Tocotrienols are naturally found in edible oils such as palm oil, annatto oil, rice bran oil, and coconut oil [10]. A recent meta-analysis of 26 clinical trials showed that vitamin E supplementation significantly reduced serum C-reactive protein (CRP) levels [12]. However, this meta-analysis included studies investigating both tocopherols and tocotrienols, and subgroup analysis specific to tocotrienols was not performed. Thus, the anti-inflammatory effect of tocotrienols could not be confirmed. In addition, there has been no systematic review examining the clinical evidence on the antioxidant effects of tocotrienols. Therefore, the present systematic review aimed to evaluate the effects of tocotrienols supplementation compared to placebo on inflammatory and oxidative stress biomarkers.

## Methods

### Study protocol

This systematic review was conducted according to the Preferred Reporting Items for Systematic Reviews and Meta-Analyses [13], and the protocol for this systematic review was registered on PROSPERO (CRD42020198241).

### Search strategy

We performed a comprehensive search in PubMed, Scopus, and Cochrane Central Register of Controlled Trials on 13 July 2020 to identify relevant studies. Medical Subject Headings terms and free-text terms for *randomized controlled trials* and *tocotrienols* were used as the search strategy (S1 Table). We did not apply keywords specific to biomarkers of inflammation and oxidative stress in the search strategy because we intended to search for all available randomized controlled trials investigating the effects of tocotrienols supplementation. We also manually searched for potential articles by checking the reference lists of relevant original articles, narrative reviews, systematic reviews, and meta-analyses from inception to July 2020.

### Study inclusion and exclusion criteria

Randomized controlled trials (parallel or crossover) were eligible for inclusion in the current systematic review if they (i) compared tocotrienols supplementation versus placebo, (ii) had an intervention for at least two weeks, and (iii) reported at least one blood biomarker of inflammation or oxidative stress before and after administration of tocotrienols. We excluded (i) *in vitro* or animal studies, (ii) non-randomized or single-arm studies, (iii) *in vivo* acute or postprandial studies, (iv) studies with combinations of tocotrienols and other dietary components, (v) studies reporting outcome measures such as urinary markers, gene expression, or *in vitro* stimulated inflammatory response, (vi) unpublished articles, abstracts, conference proceedings, or letters, and (vii) non-English publications. We screened for duplicate publications (articles based on the same dataset) through the trial registration number, list of authors, subjects’ baseline characteristics, and funder. We only included the duplicate publication with the larger subject number or longer duration of intervention.

### Study selection and data extraction

Citations from the initial search results of each database were exported to EndNote (version X7.5.3, Clarivate Analytics, Philadelphia, PA, USA), and duplicates were removed. The titles and abstracts were screened and reviewed by two authors (B-H.K. and H-C.T.). Then, full texts of potential studies were retrieved and independently reviewed in detail for inclusion based on the pre-determined criteria. Discrepancies between two authors were resolved by discussion, and a third author (A.H.A.G.) was referred if consensus could not be reached.

One author (H-C.T.) extracted the data from the included studies into a piloted sheet, and another author (B-H.K) crosschecked the extracted data. The following data were extracted: study characteristics (country, sample size, and design), subjects’ characteristics (age, sex, and population), intervention (type, dosage, duration, and placebo), biomarkers of inflammation and oxidative stress (baseline, post-intervention, and/or changes between baseline and post-intervention), and study funders. We only included data of the longest follow-up for studies with multiple time points of follow-up.

Three studies had incomplete information to derive the mean difference, and four studies presented the data in median (interquartile range) or figures. Therefore, we contacted the authors of these seven studies for data requests. However, the authors of a study published in 1996 were unreachable. Thus the study was excluded from the quantitative analysis. Out of six authors contacted, five authors had provided us with the necessary data, while one author could not respond to our request. Therefore, we used Plot Digitizer (http://plotdigitizer.sourceforge.net) to extract the data that was presented in a bar chart. Data extracted using this software was 74% agreement with the original data [14].

### Assessment of risk of bias

The risk of bias of all included studies was assessed independently by two authors (B-H.K. and H-C.T.) using The Cochrane Collaboration’s tool for assessing the risk of bias in randomized trials [15], which consisted of the following domains: random sequence generation, allocation concealment, blinding of participants and personnel, blinding of outcome assessment, incomplete outcome data, selective reporting, and other biases.

### Statistical analyses

Meta-analyses were performed for biomarkers of inflammation and oxidative stress reported by at least three studies, while a descriptive summary of findings was presented for other biomarkers. The weighted mean differences (WMD) with corresponding 95% confidence intervals (CI) were determined by pooling eligible studies for meta-analyses. Heterogeneity among the studies was assessed using the chi-squared and *I*^2^ statistics, whereby a *p*-value < 0.1 and *I*^2^ above 50% indicates having significant heterogeneity, and a random effect model was used. We also performed subgroup analysis to explore the potential source of heterogeneity among studies, according to sample size (≥ 60 or less), duration of the intervention (≥ 6 months or less), and tocotrienols dosage (≥ 400 mg/day or less). A sensitivity analysis was performed by sequentially omitting one study at a time to verify that any single study did not influence the overall result. Publication bias was assessed by using Begg’s and Egger’s tests, and visually inspecting the symmetry of the funnel plot. STATA software (version 16.0, StataCorp, College Station, TX, USA) was used for the analysis.

## Results

### The flow of study selection

**Fig 1:**
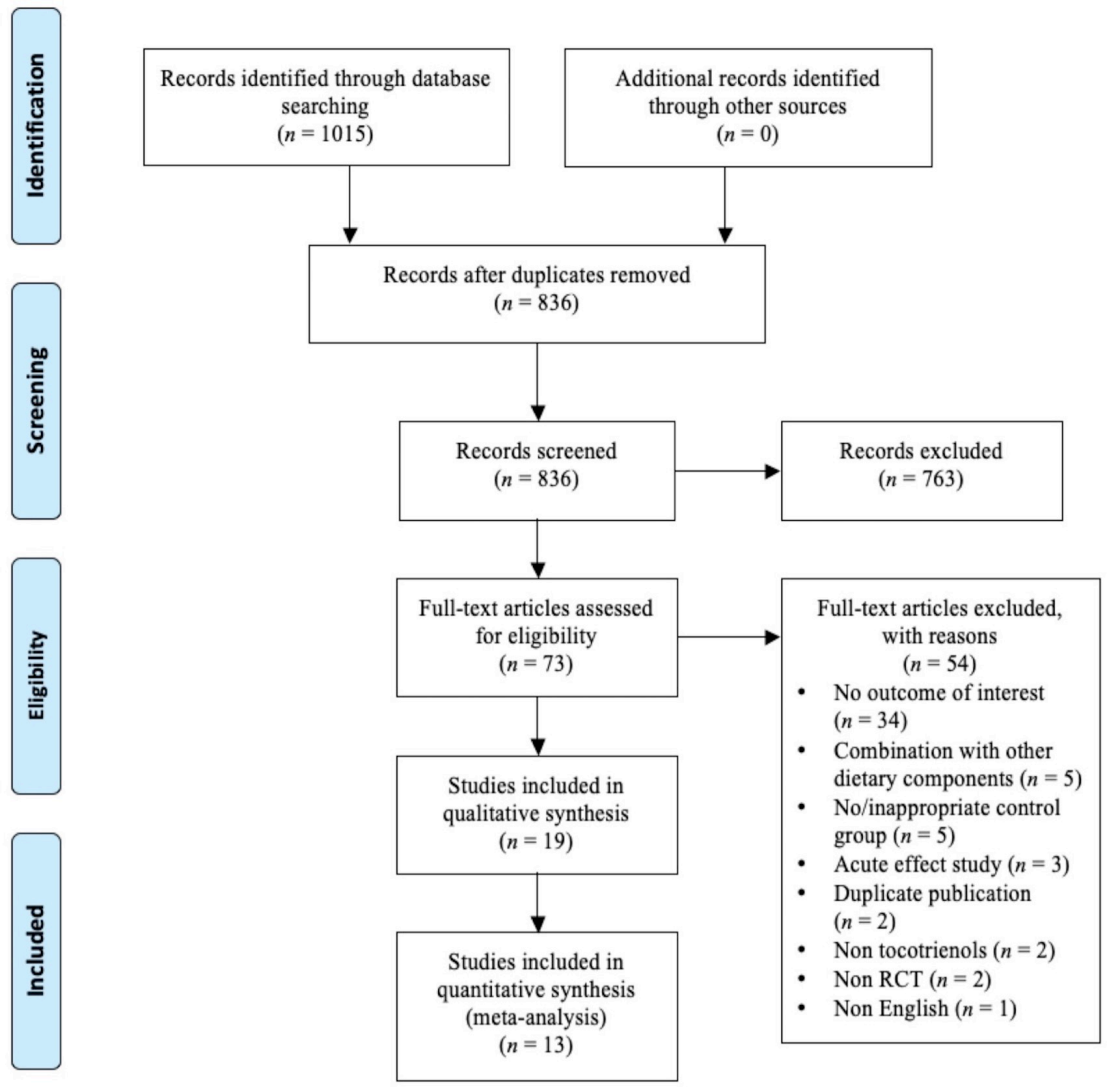
Flow diagram of study selection.

The flowchart of study selection is presented in Fig 1. From the literature search, 836 unique citations were identified from three databases. After screening the title and abstract, 73 studies were retrieved for full-text review. From this, 54 studies were excluded, and the reasons for exclusion are presented in the S2 Table. Out of the 19 studies [16–34] included in the present systematic review, there were four studies [16,17,22,23] involving subjects from two similar datasets, but these studies were included because each study reported unique biomarkers. For other duplicate publications, Pervez et al. [30] was selected due to a larger sample size, while Kooyenga et al. [25] was selected due to a longer follow-up duration. Thirteen studies [17–24,26,28,30,32,34] reporting CRP or high sensitivity C-reactive protein (hsCRP), interleukin-6 (IL-6), tumour necrosis factor-alpha (TNF-α), or malondialdehyde (MDA) were included for meta-analyses.

### Study characteristics

The characteristics of 19 studies included in the systematic review are presented in Table 1. There were 17 parallel studies [16–19,21–26,28–34] and two crossover studies [20,27]. Majority of these studies were carried out in Malaysia (*n* = 12) [16–18,20,21,24,26–28,31,33,34], followed by United States of America (*n* = 3) [19,25,29], Iran (*n* = 2) [22,23], Pakistan (*n* = 1) [30] and Australia (*n* = 1) [32]. The sample size ranged from 31 to 121 subjects, with only one study [21] having sample size above 100 subjects. The mean age of subjects ranged from 23.3 to 66.6 years old. The dose of tocotrienols supplement ranged from 80 to 600 mg/day and the details are presented in S3 Table. The intervention duration ranged from 0.5 month to 24 months, and only three studies provided tocotrienols supplements for at least 12 months [21,25,26] while the remaining studies provided intervention for 6 months or less. Most studies (*n* = 7) recruited patients with type 2 diabetes mellitus [22,23,27,28,32–34], followed by healthy individuals (*n* = 4) [16–18,31], metabolic syndrome (*n* = 2) [20,24], hypercholesterolemia (*n* = 1) [29], non-alcoholic fatty liver disease (*n* = 1) [30], hypercholesterolemia and non-alcoholic fatty liver disease (*n* = 1) [26], white matter lesion (*n* = 1) [21], end-stage kidney disease on hemodialysis (*n* = 1) [19], and coronary artery disease (*n* = 1) [25]. For inflammatory markers, eight studies reported CRP or hsCRP [19–22,24,26,30,32], four studies reported IL-6 [19,24,30,32], and three studies reported TNF-α [24,30,32]. For oxidative stress markers, seven studies reported MDA [17,18,22,27,28,30,34], two studies each reported advanced glycation end-products (AGEs) [18,33], protein carbonyl (PC) [17,18], and thiobarbituric acid reactive substances (TBARS) [19,25], respectively, and one study each reported low-density lipoprotein (LDL) oxidation status [29] and lipid oxidation products (LOPs) [25], respectively. For antioxidant enzyme activities and response, three studies reported plasma antioxidant response [19,22,31], two studies [16,18] reported erythrocyte superoxide dismutase (SOD) [16,18], catalase (CAT), and glutathione peroxidase (GPx), and one study [16] reported erythrocyte glutathione (GSH). Majority of the studies (*n* = 13) were funded by the Malaysian government or agencies [16–21,24,26–28,31–34], two studies were funded by an Iranian university [22,23], one study each was funded by a German company [29] and government of Pakistan [30], respectively, one study did not provide information on the study funder [25].

**Table 1:** Characteristics of studies and subjects included in this systematic review.

No.	Author, year	Country	*n*	Sex (M/F)	Mean Age (yr)	Population	Design	Duration (month)	T3 type (dosage)*	Placebo	Biomarkers	Funder
1.	Azman, 2018 [16], Goon 2017 [17]	Malaysia	47	17/30	52.8	Healthy	Parallel	6	TRF (150 mg/d)	Olive oil	SOD, CAT, GPx, MDA, PC, GSH	UKM
2.	Chin, 2011 [18]	Malaysia	62	0/62	≥ 35	Healthy	Parallel	6	TRF (160 mg/d)	Palm oil	SOD, CAT, GPx, PC, AGE, MDA	Malaysian government & UKM
3.	Daud, 2013 [19]	USA	81	43/38	58.5	HD	Parallel	4	TRF (180 mg/d)	Wheat germ oil	CRP, IL-6, TAP, TBARS	MPOC
4.	Gan, 2017 [20]	Malaysia	31	15/16	37.9	Metabolic syndrome	Cross-over	0.5	PTT (400 mg/d)	Palm olein	hsCRP	MBOB
5.	Gopalan, 2014 [21]	Malaysia	121	48/73	52.0	Subjects with WML	Parallel	24	Mixed T3 (400 mg/d)	Palm olein	hsCRP	MPOB
6.	Haghighat, 2013 [22], Vafa, 2015 [23]	Iran	45	22/33	55.6	T2DM	Parallel	2	T3-enriched canola oil (200 mg/d)	Canola oil	hsCRP, MDA, TAC	Tehran University of Medical Sciences
7.	Heng, 2015 [24]	Malaysia	57	20/37	41.2	Metabolic syndrome	Parallel	4	Mixed T3 (400 mg/d)	Soy bean oil	CRP, IL-6, TNF-α	Malaysian MOHE
8.	Kooyenga, 1997 [25]	USA	50	23/27	66.6	CAD	Parallel	24	α- and γ-T3 (160–240 mg/d)	Palm superolein	LOPs, TBARS	N/A
9.	Magosso, 2013 [26]	Malaysia	87	34/53	51.0	Hypercho-lesterolemic & NAFLD	Parallel	12	Mixed T3 (400 mg/d)	Placebo^†^	hsCRP	MPOB
10.	Nazaimoon, 1996 [27]	Malaysia	32	9/23	41.3	T2DM	Cross-over	6	T3 (288 mg/d)	Palm olein	MDA	MPOB
11.	Ng, 2020 [28]	Malaysia	80	52/28	63.5	T2DM	Parallel	2	T3 (400 mg/d)	Palm oil	MDA	Malaysian MOHE & MUM
12.	O’Byrne, 2000 [29]	USA	51	22/29	40.8	Hypercho-lesterolemic	Parallel	2	α- or γ- or δ- tocotrienyl acetate (250 mg/d)	MCT oil	LDL oxidation status	BASF (Germany) & NIH
13.	Pervez, 2020 [30]	Pakistan	71	34/37	44.4	NAFLD	Parallel	6	δ-T3 (600 mg/d)	Sucrose	hsCRP, IL-6, TNF-α, MDA	Government of Pakistan
14.	Rasool, 2006 [31]	Malaysia	36	36/0	23.3	Healthy	Parallel	2	TRE (80–320 mg/d)	N/A^†^	TAS	Malaysian MOSTI
15.	Stonehouse, 2016 [32]	Australia	57	36/21	60.8	T2DM, IFG or elevated WC	Parallel	2	TRF (420 mg/d)	Palm olein	hsCRP, IL-6, TNF-α	MPOB
16.	Tan, 2018 [33]	Malaysia	45	31/14	61.6	T2DM	Parallel	2	TRE (400 mg/d)	N/A^†^	AGE	MUM
17.	Tan, 2019 [34]	Malaysia	54	35/19	61.3	T2DM	Parallel	3	TRE (400 mg/d)	N/A^†^	MDA	Malaysian MOHE & MUM

*Detailed composition of the supplements is presented in S3 Table

^†^ The composition of placebo was not stated.

Abbreviation: AGE, advanced glycosylation end-product, CAD, carotid artery disease, CAT, catalase, CRP, C-reactive protein, F, female, GPx, glutathione peroxidase, GSH, gluthathione, HD, hemodialysis, hsCRP, high sensitivity C-reactive protein, IFG, impaired fasting glucose, IL-6, interleukin-6, LDL, low-density lipoprotein, LOPs, lipid peroxidation products, M, male, MCT, medium chain triglyceride, MDA, malondialdehyde, MOSTI, Ministry of Science, Technology & Innovation Malaysia, MPOB, Malaysian Palm Oil Board, MPOC, Malaysian Palm Oil Council, MUM, Monash University Malaysia, N/A, not available, NAFLD, non-alcoholic fatty liver disease, NIH, National Institutes of Health, PC, protein carbonyl, PTT, palm based tocotrienols and tocopherol, SOD, superoxide dismutase, T2DM, type 2 diabetes mellitus, T3, tocotrienols, TAC, total antioxidant capacity, TAP, total antioxidant power, TAS, total antioxidant status, TBARS, thiobarbituric acid reactive substances, TNF-α, tumor necrosis factor-alpha, TRE, tocotrienol-rich vitamin E, TRF, tocotrienol-rich fraction, UKM, Universiti Kebangsaan Malaysia, USA, United States of America, WC, waist circumference, WML, white matter lesions.

### Effects of tocotrienols supplementation on markers of inflammation

**Fig 2:**
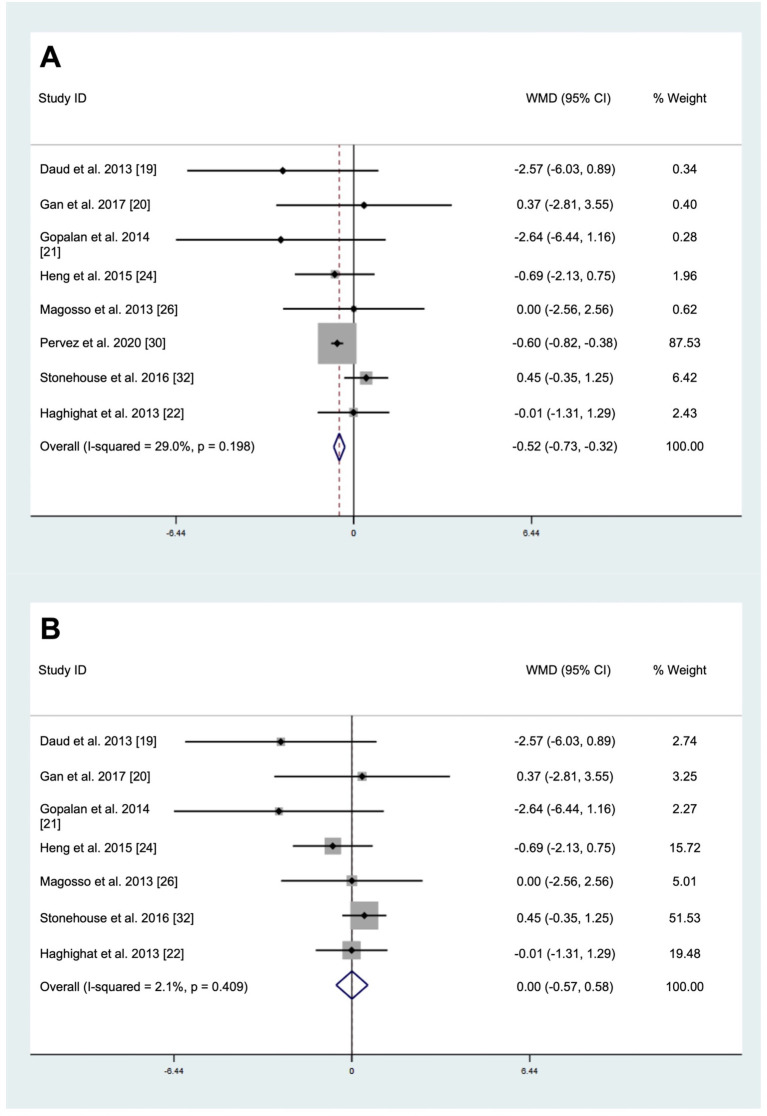
Forrest plot on the effect of tocotrienols supplementation on C-reactive protein levels for (A) analysis with all studies (B) analysis excluding Pervez et al. [30].

The pooled result of eight studies involving a total of 547 subjects (intervention = 275, placebo = 273) demonstrated a significant decrease in CRP levels after tocotrienols supplementation (WMD: -0.52 mg/L, 95% CI: -0.73, -0.32, *p* < 0.001, Fig 2) using the fixed-effect model as no significant heterogeneity was observed (*I*^2^ test = 29.0%, *p* = 0.196). Visual inspection of the funnel plot (S1 Fig), Egger’s test (*p* = 0.743), and Begg’s test (*p* = 0.458) indicated no publication bias. However, the sensitivity analysis (S2 Fig) showed that the omission of Pervez et al. [30] changed the results, indicating that the pooled result was primarily influenced by Pervez et al. [30], which accounted for 87.53% of the weightage. A separate analysis with exclusion of Pervez et al. [30] showed that supplementations of mixed tocotrienols did not affect CRP levels (WMD: 0 mg/L, 95% CI: -0.57, 0.58, *p* = 0.992, Fig 2) using the fixed-effect model as no significant heterogeneity was observed (*I*^2^ test = 2.1%, *p* = 0.409). Subgroup analyses showed that significant reductions in CRP levels were observed in study with sample size for at least 60 subjects (WMD: -0.61 mg/L, 95% CI: -0.82, -0.40, *p* < 0.001) and study for at least 6 months (WMD: -0.60 mg/L, 95% CI: -0.82, -0.39, *p* < 0.001) (Table 2).

**Table 2:** Subgroup analyses of tocotrienols on C-reactive protein level.

	*n*	WMD (95% CI)	*p* within group	*p* heterogeneity	*I*^2^ (%)
Study sample size					
≥ 60	4	-0.61 (-0.82, -0.40)	< 0.001	0.465	0
< 60	4	0.15 (-0.46, 0.75)	0.630	0.589	0
Study duration					
≥ 6 months	3	-0.60 (-0.82, -0.39)	< 0.001	0.517	0
< 6 months	5	0.07 (-0.53, 0.66)	0.822	0.377	5.3

Abbreviation: CI, confidence interval, WMD, weighted mean difference.

**Fig 3:**
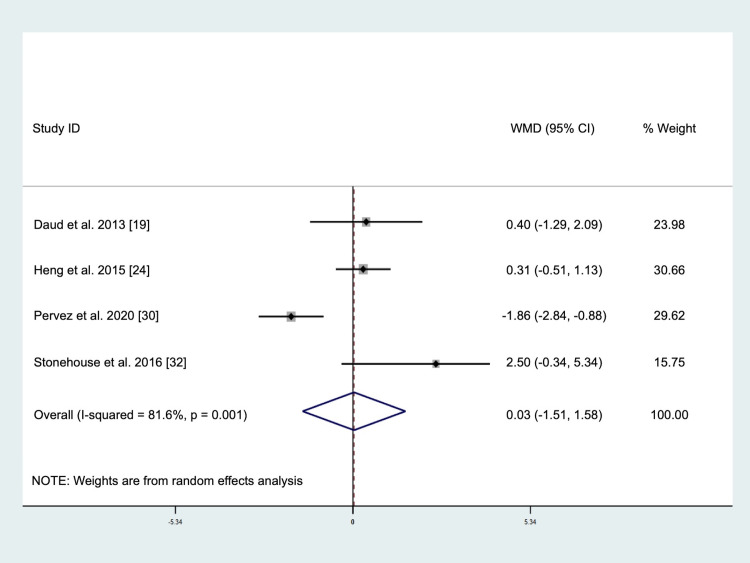
Forrest plot on the effect of tocotrienols supplementation on interleukin-6 levels.

The pooled result of four studies involving a total of 261 subjects (intervention = 132, placebo = 129) demonstrated no significant changes in IL-6 levels after tocotrienols supplementation (WMD: 0.03 pg/mL, 95% CI: -1.51, 1.58, *p* = 0.966, Fig 3) using the random-effect model due to significant heterogeneity (*I*^2^ test = 81.6%, *p* = 0.001). Visual inspection of the funnel plot (S3 Fig), Egger’s test (*p* = 0.571), and Begg’s test (*p* = 1.000) indicated that there was no publication bias. The sensitivity analysis (S4 Fig) showed that omission of Daud et al. [19] or Stonehouse et al. [32] changed the direction of effect, but the result remained non-significant, suggesting that the finding was robust. Subgroup analysis was not performed for this outcome measure.

**Fig 4:**
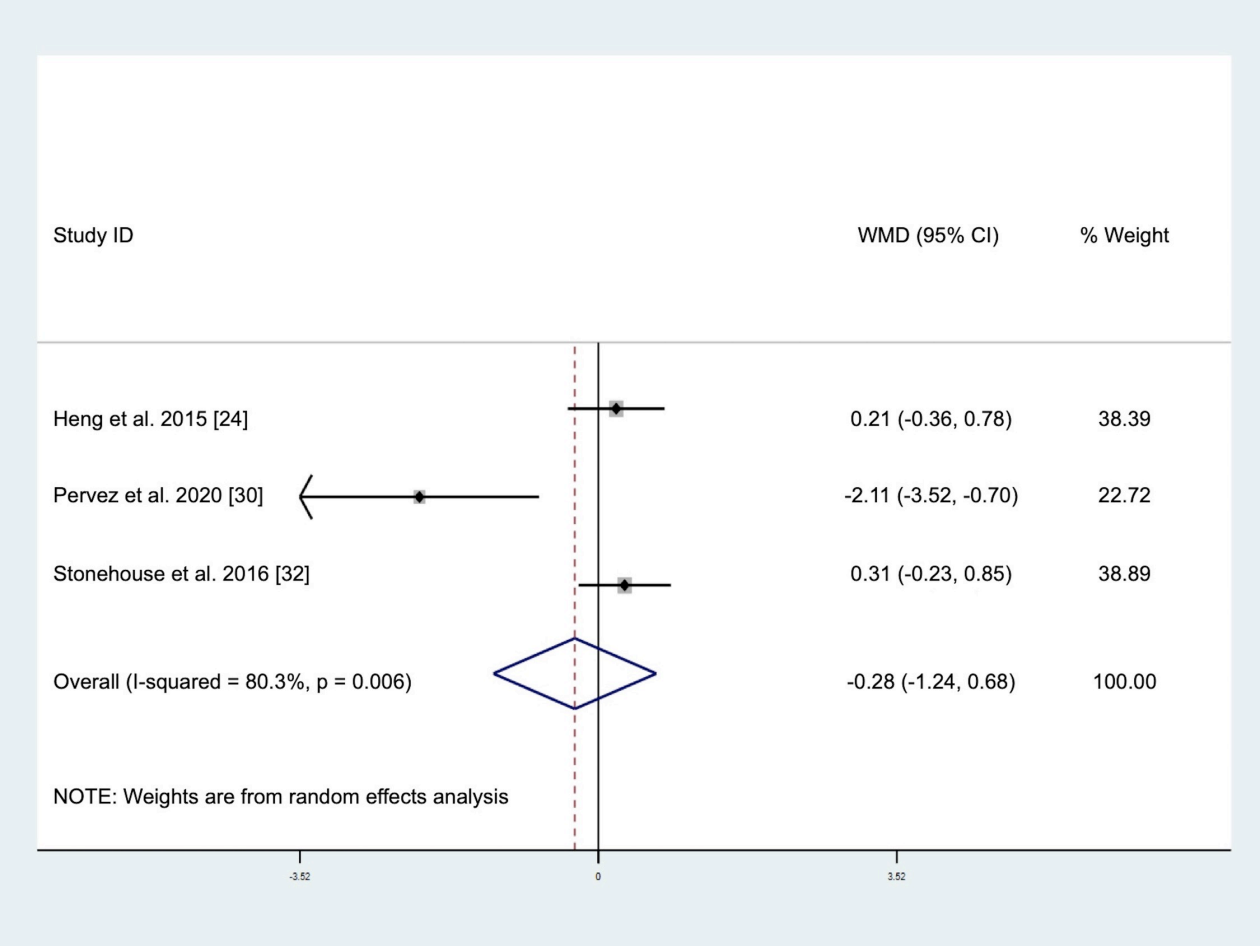
Forrest plot on the effect of tocotrienols supplementation on tumor necrosis factor-alpha levels.

The pooled result of three studies involving a total of 185 subjects (intervention = 92, placebo = 93) demonstrated no significant changes in TNF-α levels after tocotrienols supplementation (WMD: -0.28 pg/mL, 95% CI: -1.24, 0.68, *p* = 0.571, Fig 4) using the random-effect model due to significant heterogeneity (*I*^2^ test = 80.3%, *p* = 0.006). Visual inspection of the funnel plot (S5 Fig) and Begg’s test (*p* = 0.117) indicated that there was no publication bias. Although Egger’s test (*p* = 0.011) was < 0.05, the trim-and-fill analysis did not identify any missing studies, suggesting a possible absence of bias. The sensitivity analysis (S6 Fig) showed that the omission of Pervez et al. [30] changed the direction of effect, but the result remained not significant, suggesting that the finding was robust. Subgroup analysis was not performed for this outcome measure.

### Effects of tocotrienols supplementation on oxidative stress markers

**Fig 5:**
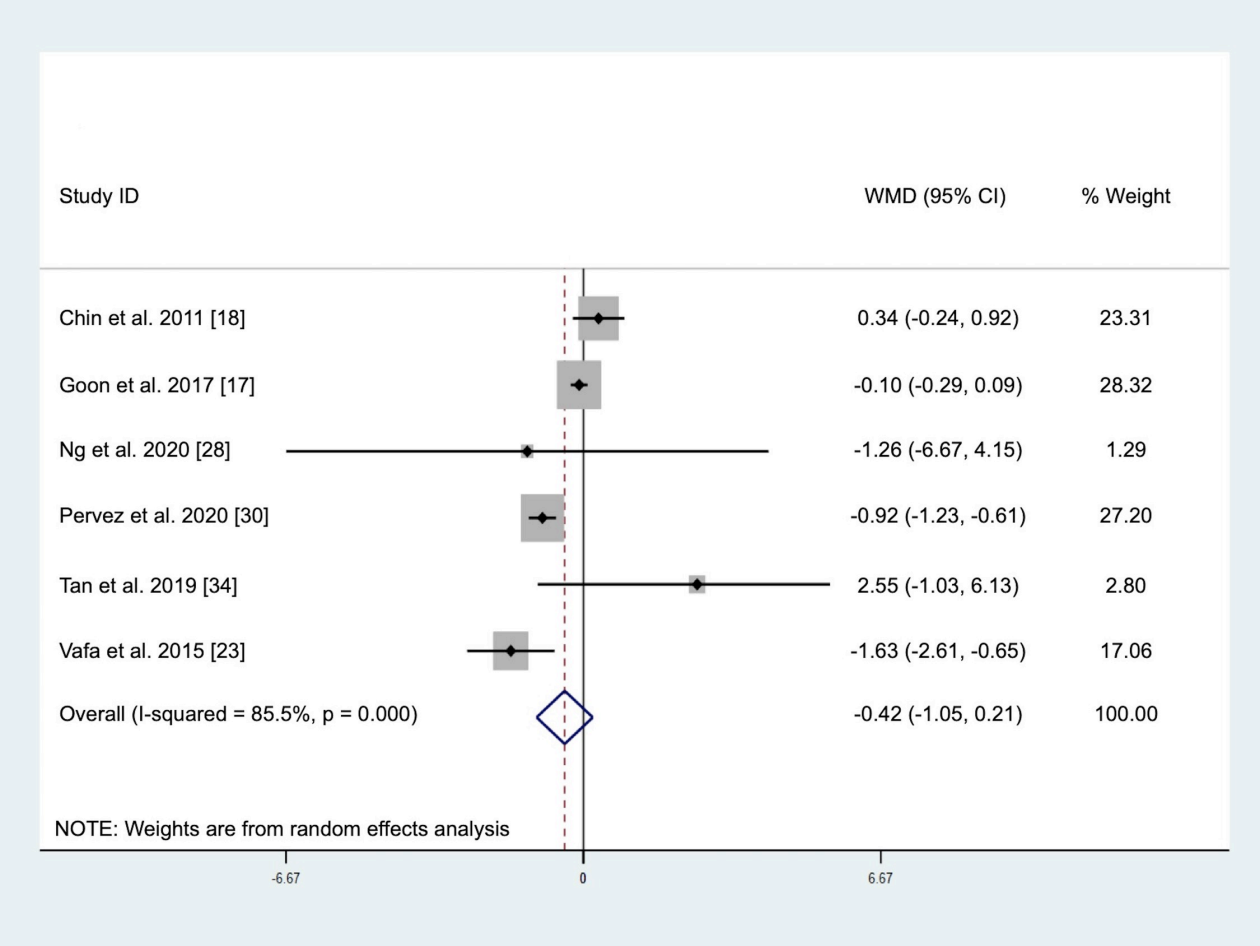
Forrest plot on the effect of tocotrienols supplementation on malondialdehyde levels.

The pooled result of six studies involving a total of 356 subjects (intervention = 179, placebo = 177) demonstrated no significant changes in MDA levels after tocotrienols supplementation (WMD: -0.42 μmol/L, 95% CI: -1.05, 0.21, *p* = 0.189, Fig 5) using the random-effect model due to significant heterogeneity (*I*^2^ test = 85.5%, *p* < 0.001). Visual inspection of the funnel plot (S7 Fig), Egger’s test (*p* = 0.844), and Begg’s test (*p* = 0.573) indicated an absence of publication bias. The sensitivity analysis (S8 Fig) showed that omission of any study did not change the finding, suggesting that the result was robust. Subgroup analyses showed that the effect of tocotrienols on MDA levels was not influenced by study sample size or duration (Table 3). However, a significant reduction in MDA levels was observed in studies with tocotrienols dosage provided at least 400 mg/day (WMD: -0.90 μmol/L, 95% CI: -1.20, -0.59, *p* < 0.001).

**Table 3:** Subgroup analyses of tocotrienols on malondialdehyde level.

	*n*	WMD (95% CI)	*p* within group	*p* heterogeneity	*I*^2^ (%)
Study sample size					
≥ 60	3	-0.36 (-1.52, 0.80)	0.546	0.001	86.0
< 60	3	-0.38 (-1.83, 1.06)	0.602	0.004	82.2
Study duration					
≥ 6 months	3	-0.26 (-0.91, 0.40)	0.444	< 0.001	91.9
< 6 months	3	0.34 (-3.13, 2.46)	0.812	0.087	59.0
Dosage					
≥ 400 mg/day	3	-0.90 (-1.20, -0.59)	< 0.001	0.165	44.6
< 400 mg/day	3	-0.33 (-1.08, 0.42)	0.393	0.003	82.7

Abbreviation: CI, confidence interval, WMD, weighted mean difference.

The effects of tocotrienols supplementation on oxidative stress markers are summarized in Table 4. Chin et al. [18] observed significant reductions in PC concentrations in subjects after receiving tocotrienols supplement (baseline: 0.63 ± 0.04 nmol/mg, 6^th^ month: 0.45 ± 0.04 nmol/mg, *p* < 0.01), and subgroup analyses showed that the significant reduction was only observed among subjects ≥ 50 years old (baseline: 0.63 ± 0.03 nmol/mg, 6^th^ month: 0.40 ± 0.03 nmol/mg, *p* < 0.01), but not 35–49 years old. Contrarily, Goon et al. [17] did not observe any significant change in PC concentrations in subjects receiving tocotrienols supplement (baseline: 0.44 ± 0.04 nmol/mg, 6^th^ month: 0.33 ± 0.03 nmol/mg, *p* > 0.05).

**Table 4:** Descriptive summary of the effects of tocotrienols supplementation on oxidative stress markers, antioxidant enzyme activities and antioxidant response.

Biomarkers	Summary of findings and reference
PC	↓ [18], ↔ [17]
AGEs	↔ [18,33]
TBARS	↓ [25], ↔ [19]
LOPs	↔ [25]
LDL oxidation rate	↓ [29]
SOD	↓ [18], ↑ [16]
CAT	↔ [16,18]
GPx	↔ [16,18]
GSH	↔ [16]
Antioxidant response	↑ TAS (320 mg/day) [31], ↔ TAP or TAC [19,23]

Symbol: ↓, significant reduction, ↑, significant increase, ↔, no effect.

Abbreviations: AGEs, advanced glycation end-products, CAT, catalase, GPx, glutathione peroxidase, GSH, glutathione, LDL, low density lipoprotein, LOPs, lipid peroxidation products, PC, protein carbonyl, SOD, superoxide dismutase, TAC, total antioxidant capacity, TAP, total antioxidant power, TAS, total antioxidant status, TBARS, thiobarbituric acid reactive substances.

For AGEs, Chin et al. [18] showed that tocotrienols supplementation for 6 months did not lower serum AGE levels in all subjects (baseline: 2.38 ± 0.19 units/ml, 6^th^ month: 1.92 ± 0.14 units/ml, *p* > 0.05), but a significant reduction in serum AGE level for subjects above 50 years old (baseline: 2.73 ± 0.26 units/ml, 6^th^ month: 1.71 ± 0.24 units/ml, *p* < 0.05) was observed. On the other hand, Tan et al. [33] showed that changes in serum AGE levels were not significant between patients with type 2 diabetes mellitus receiving placebo or tocotrienols supplement (placebo: 83.7 ± 27.2 μg/mL, tocotrienols: 89.8 ± 27.2 μg/mL, *p* = 0.874).

Kooyenga et al. [25] reported significant reductions in TBARS levels in subjects after receiving tocotrienols (Baseline: 1.08 ± 0.14 μM, 24^th^ month: 0.80 ± 0.14 μM, *p* < 0.05). Contrarily, Daud et al. [19] did not observe any significant change in TBARS levels with tocotrienols supplementations (baseline: 3.01 ± 4.65 μM MDA, 4^th^ month: 2.89 ± 3.65 μM MDA, *p* > 0.05), though the tocotrienols group had a marginally (*p* = 0.055) lower trend in TBARS levels at the 4^th^ month compared to the placebo group.

Kooyenga et al. [25] reported that the changes in LOPS levels were not significant in subjects after receiving tocotrienols supplementations (baseline: 1.73 ± 0.15 μM, 24^th^ month 2.85 ± 0.55 μM, *p* > 0.05). Byrne et al. [29] compared the effects of three different tocotrienols isomers on LDL oxidative profile and showed significant reductions in the rate of LDL oxidation in subjects receiving α-tocotrienols (difference: -0.6 ± 0.9 nmol/mg/min, *p* <0.01) or δ-tocotrienols (difference: -0.1 ± 1.1 nmol/mg/min, *p* <0.06), but not γ-tocotrienols and placebo.

### Effects of tocotrienols supplementation on antioxidant enzyme activities and response

The effects of tocotrienols supplementation on antioxidant enzyme activities and response are summarized in Table 4. There was a discrepancy in the finding of the effect of tocotrienols supplements on erythrocyte SOD levels [16,18], while no significant changes in erythrocyte CAT, GPx, and GSH levels following tocotrienols supplementation were observed. One study reported a significant increase in total antioxidant status with tocotrienols supplement [31], and this effect was not reported by the other two studies [19,23].

### Risk of bias assessment

**Fig 6:**
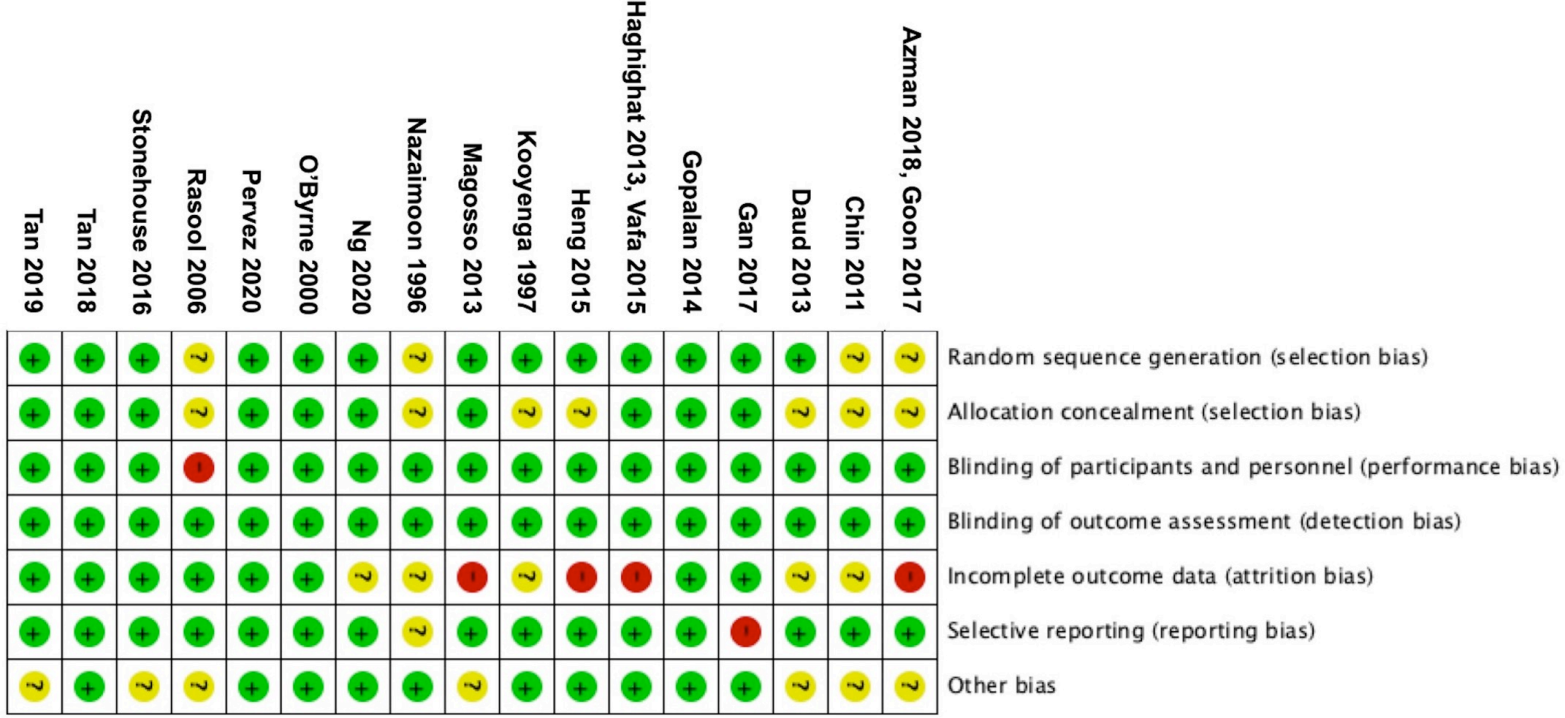
Risk of bias summary.

**Fig 7:**
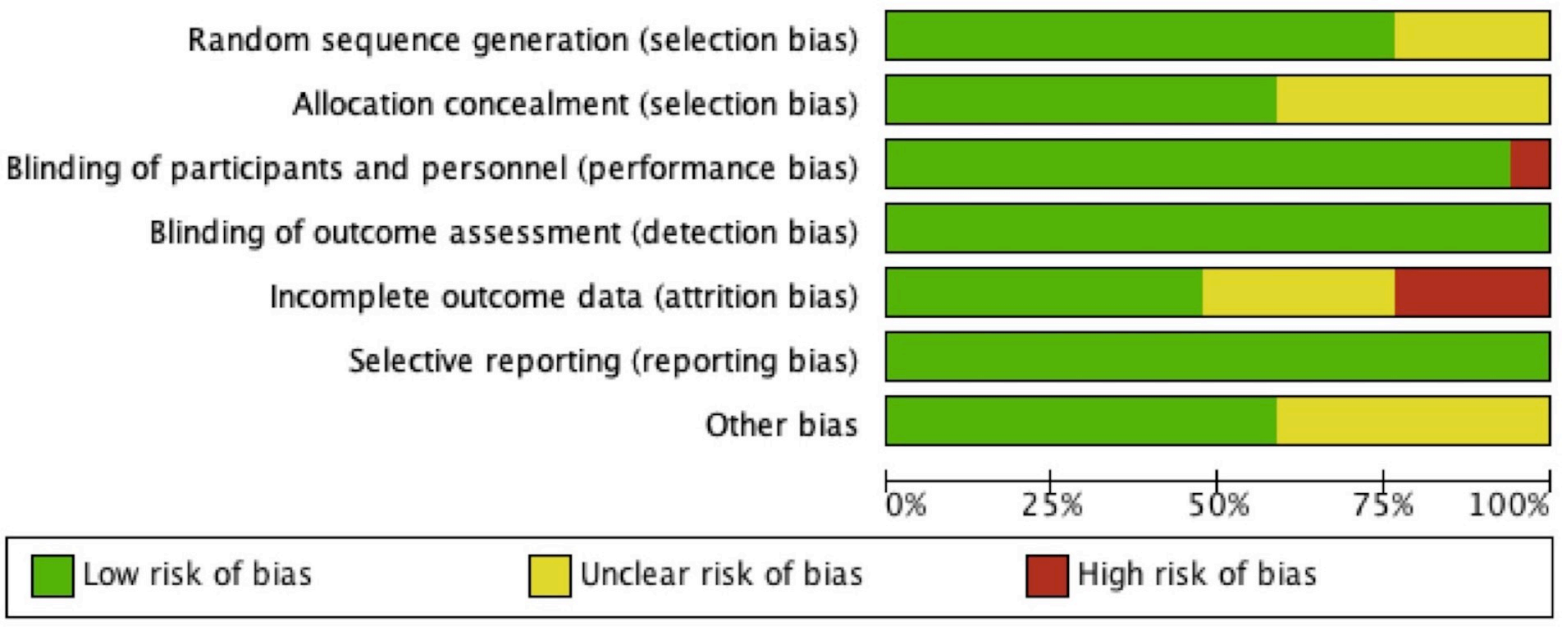
Risk of bias graph.

The bias assessment of the included studies is presented in **Figs 6** and **7**. A high risk of bias related to incomplete outcome data (attrition bias) was identified in six studies, which applied the “per-protocol” or “as-treated” analyses, while the other five studies had insufficient information to permit judgment. There were eight studies with unclear risk of selection bias concerning either random sequence generation or allocation concealment. One study had a blinded end-point design, which was considered high risk for performance bias. All studies were considered to have a low risk for detection bias as research personnels were blinded, and outcomes of this systematic review were objective markers and unlikely influenced by assessors. One study was considered to have a high risk for reporting bias due to a lack of baseline data. Seven studies had unclear risk of other biases, as there were potential risks of bias due to baseline characteristic imbalance or presence of potential confounding factors affecting the outcomes.

## Discussion

To our knowledge, this is the first systematic review and meta-analysis of randomized controlled trials to evaluate the effects of tocotrienols supplementation on circulating biomarkers of inflammation and oxidative stress. We demonstrated that tocotrienols supplementation reduced CRP levels. However, this finding needs to be interpreted with caution as the sensitivity analysis showed that the result was mainly driven by the study of Pervez et al. [30]. Once this study was excluded from the meta-analysis, the effects on CRP became non-significant. Pervez et al. [30] used an individual isoform of tocotrienols, namely δ-tocotrienols, while mixed tocotrienols were used by other studies [19–22,24,26,32]. In addition, the dosage of tocotrienols used by Pervez et al. [30] was 600 mg/day, while the remaining studies provided tocotrienols supplements with a dosage between 180 and 400 mg/day. Therefore, the type and dosage of tocotrienols supplementation may have contributed to the discrepancy of these findings as pooled analysis of studies supplementing mixed tocotrienols did not significantly lower CRP levels while only one study providing δ-tocotrienols showed a significant reduction in CRP levels. The subgroup analysis showed a significant reduction in CRP levels in studies with at least six months of intervention duration. For other markers, our meta-analyses demonstrated that tocotrienols supplementations had no effects on IL-6, TNF-α, and MDA levels, but the subgroup analysis showed that tocotrienols supplementation at 400 mg/day significantly lowered MDA levels. Therefore, it is suggested that future studies should consider at least providing tocotrienols supplement at 400 mg/day for six months.

Several meta-analyses have investigated the effects of vitamin E supplementation, particularly tocopherols, on inflammatory and oxidative stress outcomes. A meta-analysis of 12 studies by Saboori et al. [35] demonstrated that tocopherols supplementation was associated with significant reduction in CRP level by 0.62 mg/L (95% CI: -0.92, -0.31, *p* < 0.001). A meta-analysis of vitamin E supplementation (mainly tocopherols) in patients on hemodialysis also reported a significant reduction in CRP levels [36]. Similarly, a recent meta-analysis inclusive of studies investigating both tocopherols and tocotrienols showed a significant reduction in CRP levels, while the significant reductions in IL-6 and TNF-α were reported for α-tocopherol and γ-tocopherol, respectively [12]. It must be noted that a subgroup analysis for tocotrienols was not performed, and several studies on tocotrienols [22,24,26,30] were not included in the meta-analysis by Asbaghi et al. [12]. Contrarily, Fouladvand et al. [37] pooled the results from eight studies on vitamin C and E co-supplementation and showed no significant effect on CRP levels. For oxidative stress, Sepidarkish et al. [38] showed that omega-3 fatty acids and vitamin E co-supplementation reduced MDA levels and increased total antioxidant capacity and nitric oxide levels. However, no significant effects on GSH, CAT, and SOD were observed. The meta-analysis by Rad et al. [39] showed that vitamin E supplementation significantly reduced plasma isoprostane level but had no effect on urine isoprostane level. It is important to highlight that these findings were primarily based on tocopherols and should not be extrapolated to tocotrienols.

Pre-clinical studies have proposed several mechanisms for the anti-inflammatory properties of tocotrienols: (i) inhibition of NF-kB [40], (ii) suppressing the expression of inflammatory mediators such as TNF-α, interleukin-1, IL-6, interleukin-8, inducible nitric oxide synthase, and (iii) suppressing the STAT3 cell-signaling pathway [10]. Several preclinical studies have investigated the anti-inflammatory properties of individual tocotrienols isoforms. Yam et al. [41] compared the anti-inflammatory effects of tocotrienols rich fraction (TRF) and individual tocotrienols isoforms using the model of lipopolysaccharide (LPS)-stimulated RAW264.6 macrophages. It was demonstrated that the δ-tocotrienols had the highest inhibitory effect on the production of IL-6, followed by γ-tocotrienols, α-tocotrienols, and TRF. On the other hand, production of TNF-α was reduced in the α-tocotrienols group, unaltered in the γ-tocotrienols, and increased in the TRF and δ-tocotrienols groups. On the contrary, Qureshi et al. [42] demonstrated that δ-tocotrienols had the greatest inhibitory effect on TNF-α in LPS-stimulated RAW 264.7 cells and 6-week old female BALB/c mice, followed by γ-tocotrienols and α-tocotrienols. Muid et al. [43] showed that both δ- and γ-tocotrienols had a higher potency in terms of inhibiting IL-6 production and NF-κB activation in LPS-stimulated human umbilical vein endothelial cells than α- and β-tocotrienols. Selvaduray et al. [44] also reported that the capacity of suppressing the production of IL-6 and IL-8 in human umbilical vein endothelial cells was most significant in δ-tocotrienols, followed by γ-tocotrienols and TRF. Nishio et al. [45] showed that both the capacity of α- and γ-tocotrienols suppressing the production of reactive oxygen species and expression of TNF-α and IL-8 induced by treatment of LPS to human lung carcinoma A49 cells was not significantly different. Therefore, individual isoforms of tocotrienols have different potency of anti-inflammatory effects, and it is critical to distinguish these isomers when interpreting the findings of studies included in this systematic review.

However, the present systematic review of clinical trials could not provide conclusive clinical evidence on the anti-inflammatory and antioxidant effects of tocotrienols. Similarly, despite the accumulating evidence from *in vitro* and animal studies suggesting that tocotrienols are a potential hypocholesterolemic agent [9], a recent meta-analysis by Zuo et al. [46] also demonstrated that tocotrienols supplementation only significantly increased high-density lipoprotein-cholesterol levels but had no influence on total cholesterol, low-density lipoprotein cholesterol, or triglyceride levels. Therefore, it appeared that findings from pre-clinical studies were not entirely reproducible in human trials. Several possibilities on the challenges of clinical translation from bench to bedside merit consideration. Firstly, tocotrienols have relatively low bioavailability, and the oral absorption of tocotrienols is highly dependent on the consumption of dietary fat due to their lipid-solubility, thus ensuring bile secretion and emulsification process in the intestine to facilitate absorption [9]. Orally administered tocotrienols have shorter elimination half-time by 4.5–8.7 times compared to α-tocopherol [47]. Therefore, twice-daily dosing of tocotrienols supplement is necessary to ensure tocotrienols in the circulating plasma for a sustained period [48]. In addition, supplementation dose and formulation, subjects’ age, and underlying diseases are critical factors affecting the bioavailability and clinical efficacy of tocotrienols [49]. In the present systematic review, eight studies [16–18,21,26,29,31,32] reported a significant increase in serum tocotrienols levels, while one study reported a non-significant increase in tocotrienols level [27]. The remaining studies did not assess changes in serum tocotrienols levels.

The present systematic review included randomized controlled trials reporting a wide variety of biomarkers of inflammation, oxidative stress, and antioxidant enzyme and response with very limited common parameters for meta-analysis. The most frequently reported inflammatory biomarkers were CRP, IL-6, and TNF-α, while MDA was the most frequently reported oxidative stress marker. Inflammation and oxidative stress are biological responses orchestrated via a complex network of interactions, and these four biomarkers only represent a small part of these processes. In addition, these markers do not differentiate acute and chronic inflammation, and different phases of inflammatory responses. Measurement of isoprostanes with the mass spectrometric technique is known as the standard of oxidative stress assessment [50], yet none of the studies included in this systematic review reported it. Contrarily, antioxidant enzymes were commonly reported, albeit these markers reflected neither inflammation nor oxidative stress. Robust and validated markers are essential considerations for human nutritional studies elucidating the role of nutrition supplements such as tocotrienols in inflammation and oxidative stress. Therefore, a cluster of validated markers instead of a single marker has been recommended to evaluate inflammatory and oxidative stress status [51]. The use of new omics technologies could also shed light on the impacts of tocotrienols supplementation on inflammation and oxidative stress.

It is also important to note that these inflammatory and oxidative stress markers may fluctuate over time, even in healthy individuals, as several modifying factors influence the concentrations of these markers, including age, body fatness, physical activity, sex, genotype, smoking habits, diet, the composition of the gut microbiota and the use of certain medications [51]. For instance, individuals with chronic diseases may exhibit a greater degree of chronic inflammation than healthy individuals. In this systematic review, we included a heterogeneous group of subjects with different disease statuses. Therefore, the effective dose of tocotrienols to exert anti-inflammatory and antioxidative effects may vary by subjects’ characteristics and underlying conditions, which leads to null findings in some analyses. In the context of dietary intake, most studies included in this systematic review were conducted in Malaysia, and about 84–90% of Malaysians regularly consume palm oil [52,53], which contains a high amount of tocotrienols (940 mg/kg) [10]. Therefore, baseline and variations in dietary habits of these subjects should be taken into consideration.

Our meta-analysis had several limitations. Firstly, although 19 studies were included in the present systematic review, the number of studies analyzing specific biomarkers was small. We could not perform meta-analysis if fewer than three studies reported these oxidative stress markers and antioxidant enzyme activities. Although we performed meta-analyses for four biomarkers of inflammation and oxidative stress, these selected biomarkers do not represent the whole picture of the complex physiological process of inflammatory response and oxidative stress. Secondly, most studies had a relatively small sample size and short intervention period. Thirdly, we could not perform subgroup analyses for meta-analyses on IL-6 and TNF-α due to limited studies; therefore, the source of between-study heterogeneity cannot be identified. In addition, potential bias may exist in the group selection for the subgroup analyses. However, the subgroup analyses aimed to generate a hypothesis on the potential effects of tocotrienols based on the study characteristics that require further investigation. Fourthly, some studies may be underpowered, as markers of inflammation and oxidative stress were not designated as the primary outcome. Then, studies included in this systematic review consisted of subjects with varied underlying diseases associated with a different degree of inflammation and oxidative stress. Lastly, the external validity of our results requires further confirmation as most studies were conducted in Malaysia.

## Conclusions

The meta-analysis showed that tocotrienols supplementations significantly lowered CRP levels, but this finding was primarily attributed to a single study using δ-tocotrienols, not mixed tocotrienols. In addition, there were no significant effects on IL-6, TNF-α, and MDA levels. Although it appeared that a higher dose of tocotrienols (≥ 400 mg/day) might reduce MDA levels, further studies are required to confirm this observation. The effects of tocotrienols supplementations on other oxidative stress markers, antioxidant enzyme activities and status were inconclusive due to limited high-quality clinical evidence. More randomized controlled trials are warranted to confirm the anti-inflammatory and antioxidant effects of tocotrienols by assessing validated inflammatory and oxidative stress biomarkers determined using state-of-the-art technologies. In addition, the effects of different isoforms and dosages of tocotrienols, and populations that are most likely to benefit from the supplementation, such as populations with suboptimal baseline plasma tocotrienols concentration or known active inflammatory diseases, should also be investigated.

## Supporting information

S1 Checklist PRISMA 2009 checklist. (DOC)

S1 Fig Funnel plot of studies investigating the effects of tocotrienols supplementation on C-reactive protein. (TIF)

S2 Fig Sensitivity analysis of meta-analysis on the effect of tocotrienols supplementation on C-reactive protein. (TIF)

S3 Fig Funnel plot of studies investigating the effect of tocotrienols supplementation on interleukin-6. (TIF)

S4 Fig Sensitivity analysis of meta-analysis on the effect of tocotrienols supplementation on interleukin-6. (TIF)

S5 Fig Funnel plot of studies investigating the effect of tocotrienols supplementation on tumor necrosis factor-alpha. (TIF)

S6 Fig Sensitivity analysis of meta-analysis on the effect of tocotrienols supplementation on tumor necrosis factor-alpha. (TIF)

S7 Fig Funnel plot of studies investigating the effect of tocotrienols supplementation on malondialdehyde. (TIF)

S8 Fig Sensitivity analysis of meta-analysis on the effect of tocotrienols supplementation on malondialdehyde. (TIF)

S1 Table Full search strategy. (DOCX)

S2 Table Study selection based on inclusion criteria after reviewing full text. (DOCX)

S3 Table Additional information on the tocotrienols supplements used in all studies. (DOCX)
