# Supplementary figures and images for: Effects of tocotrienols supplementation on markers of inflammation and oxidative stress: A systematic review and meta-analysis of randomized controlled trials
Source: PLoS One. 2021 Jul 23;16(7):e0255205. doi: 10.1371/journal.pone.0255205 (PMC8301652; doi:10.1371/journal.pone.0255205)

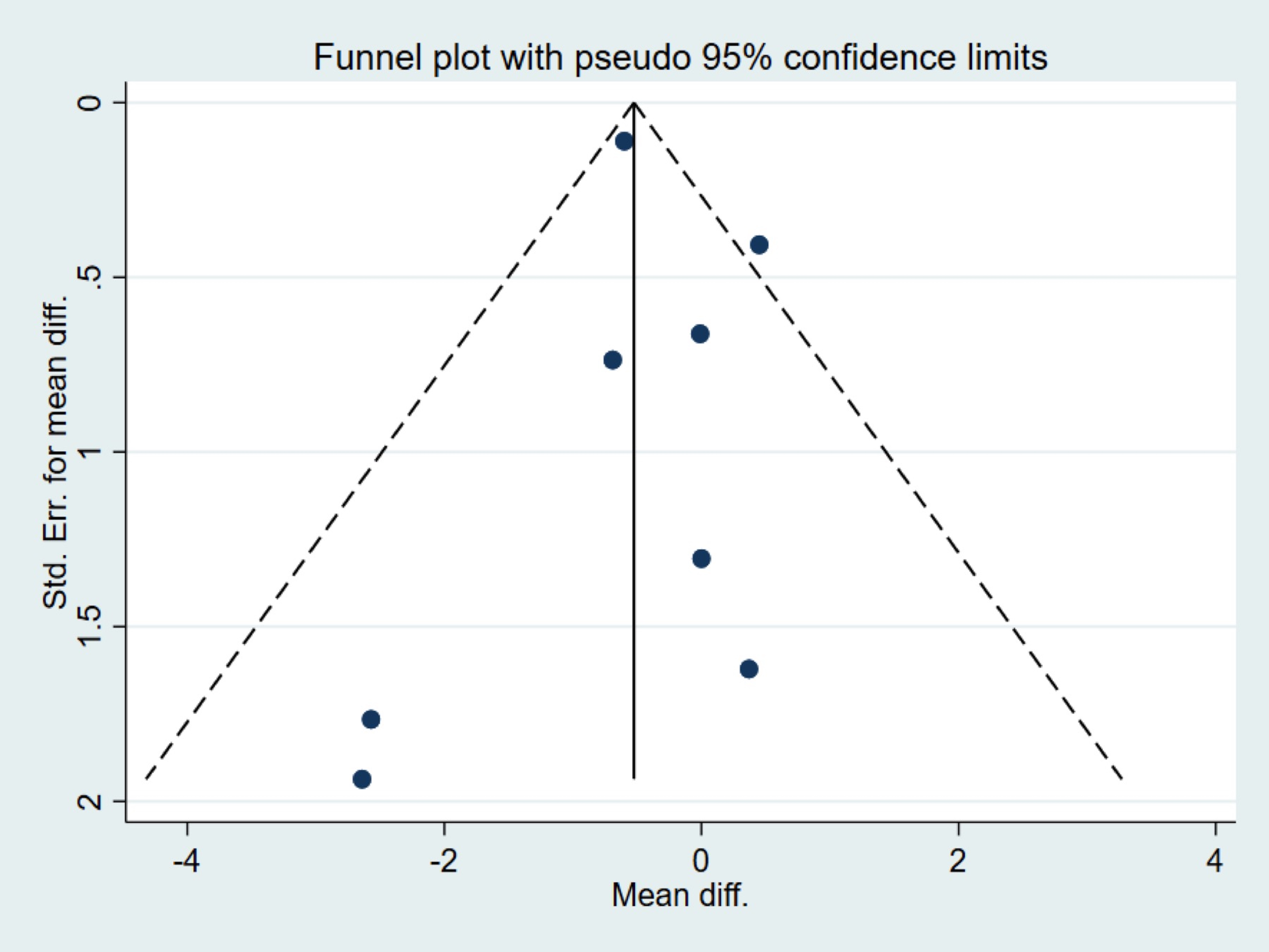

Supplement: S1 Fig — (TIF) [file pone.0255205.s002.tif]

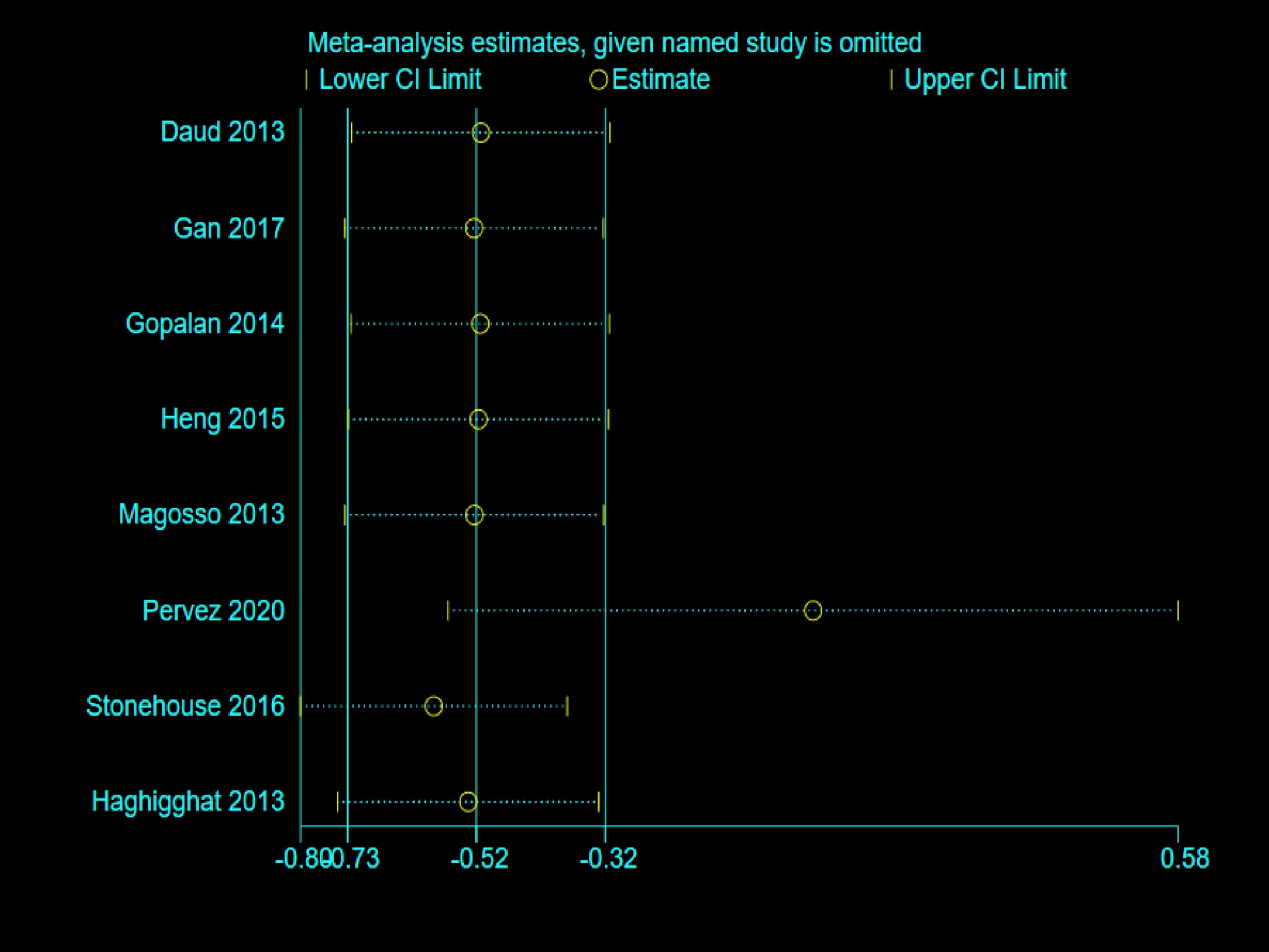

Supplement: S2 Fig — (TIF) [file pone.0255205.s003.tif]

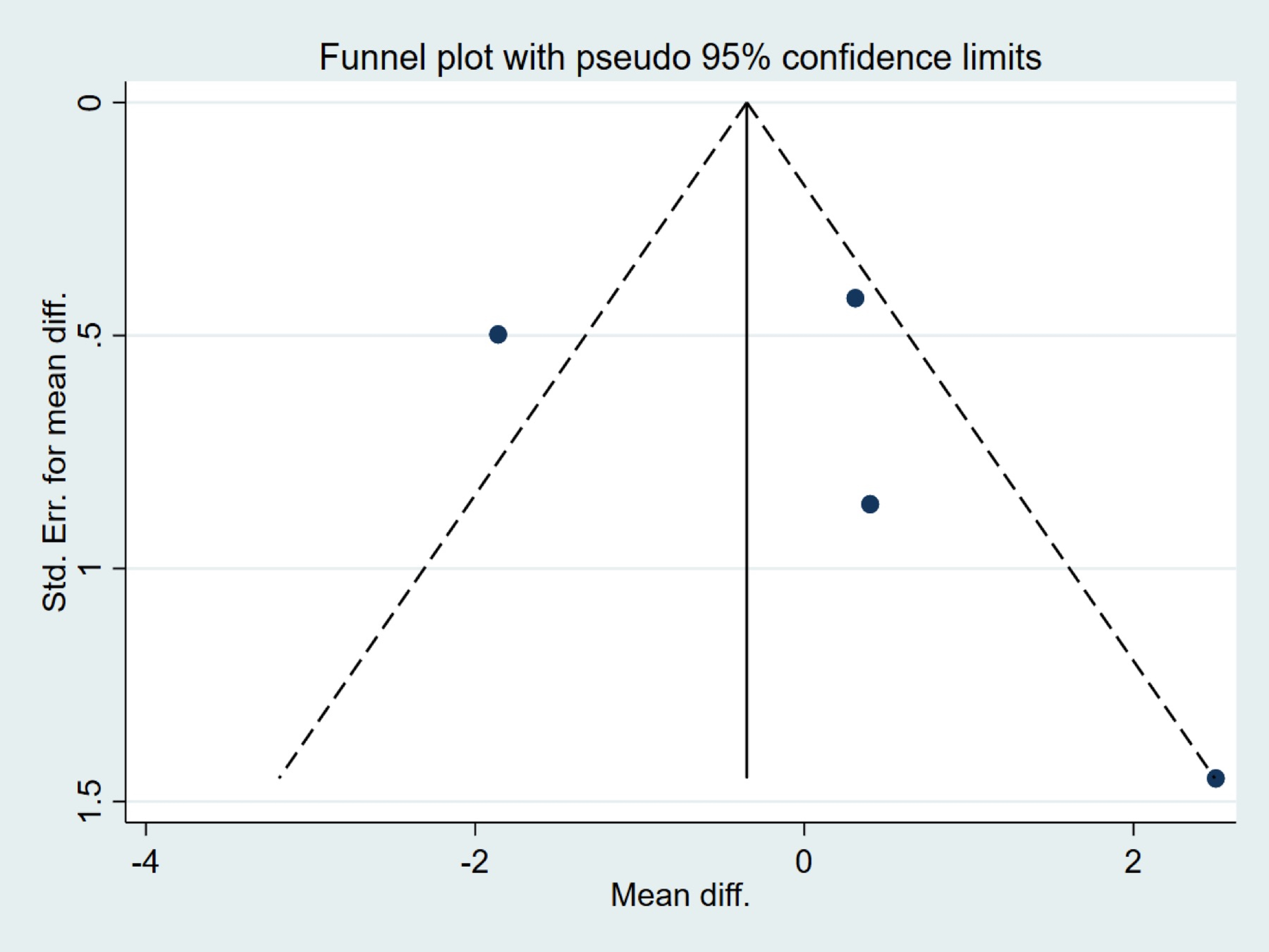

Supplement: S3 Fig — (TIF) [file pone.0255205.s004.tif]

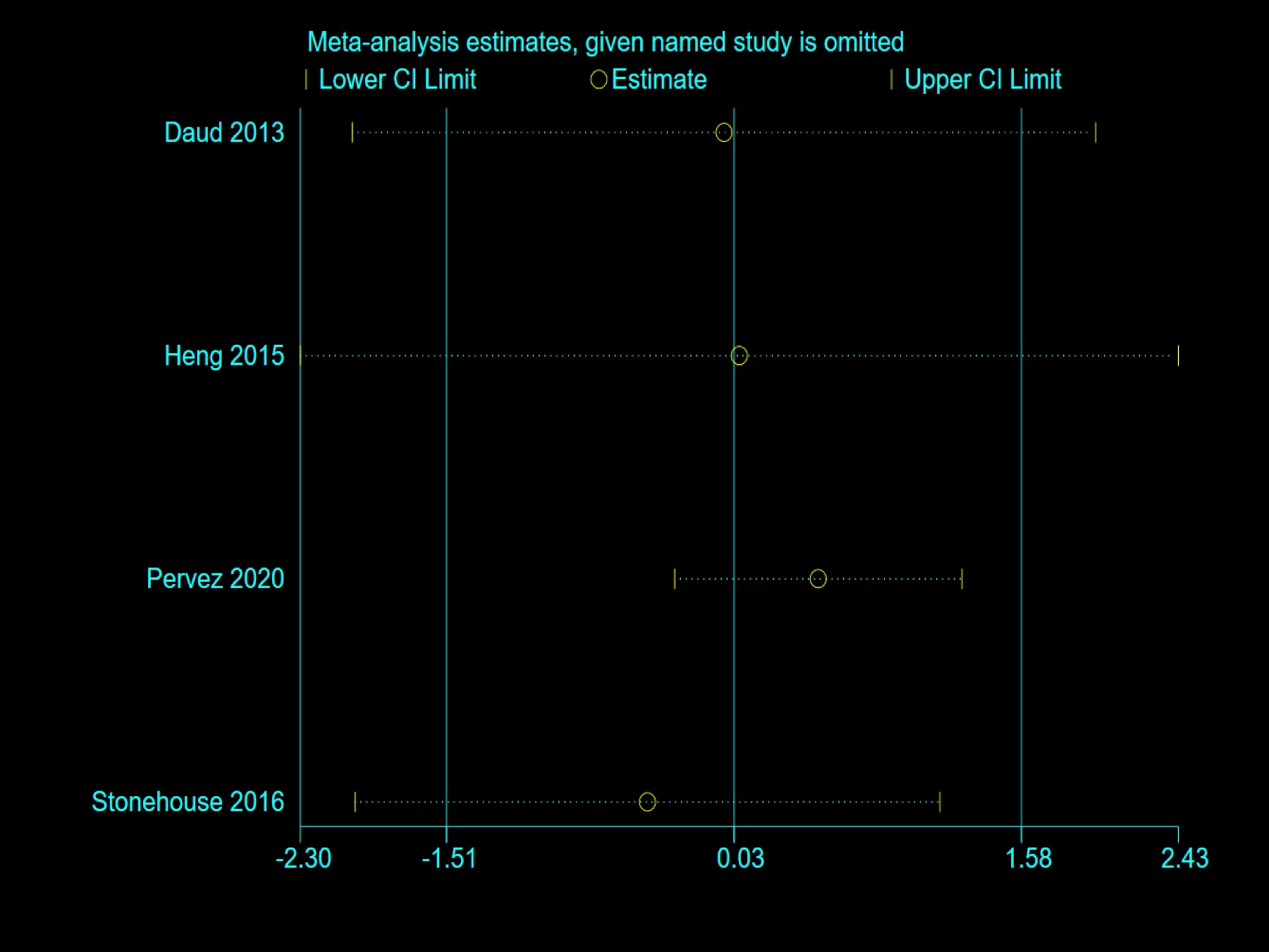

Supplement: S4 Fig — (TIF) [file pone.0255205.s005.tif]

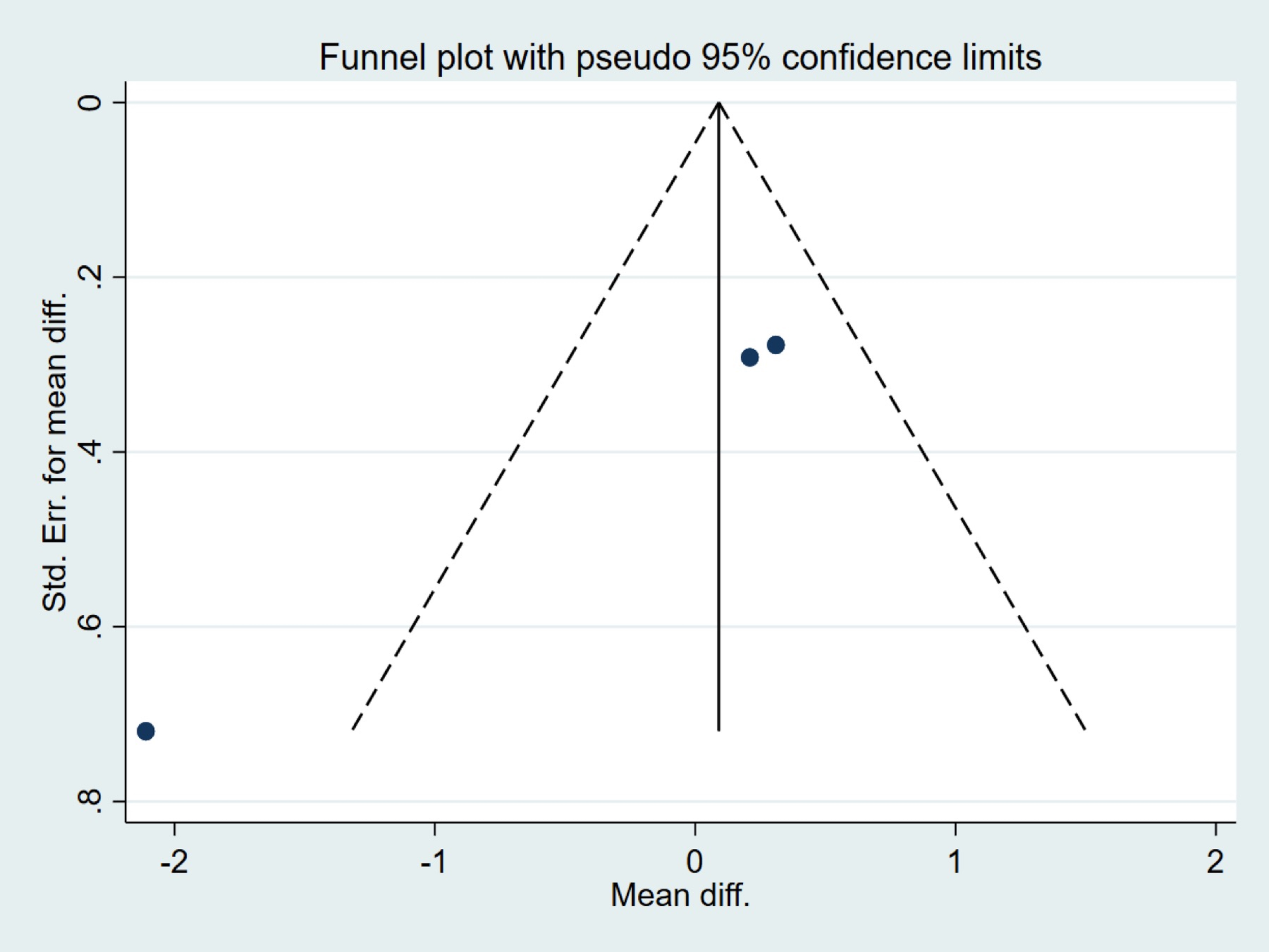

Supplement: S5 Fig — (TIF) [file pone.0255205.s006.tif]

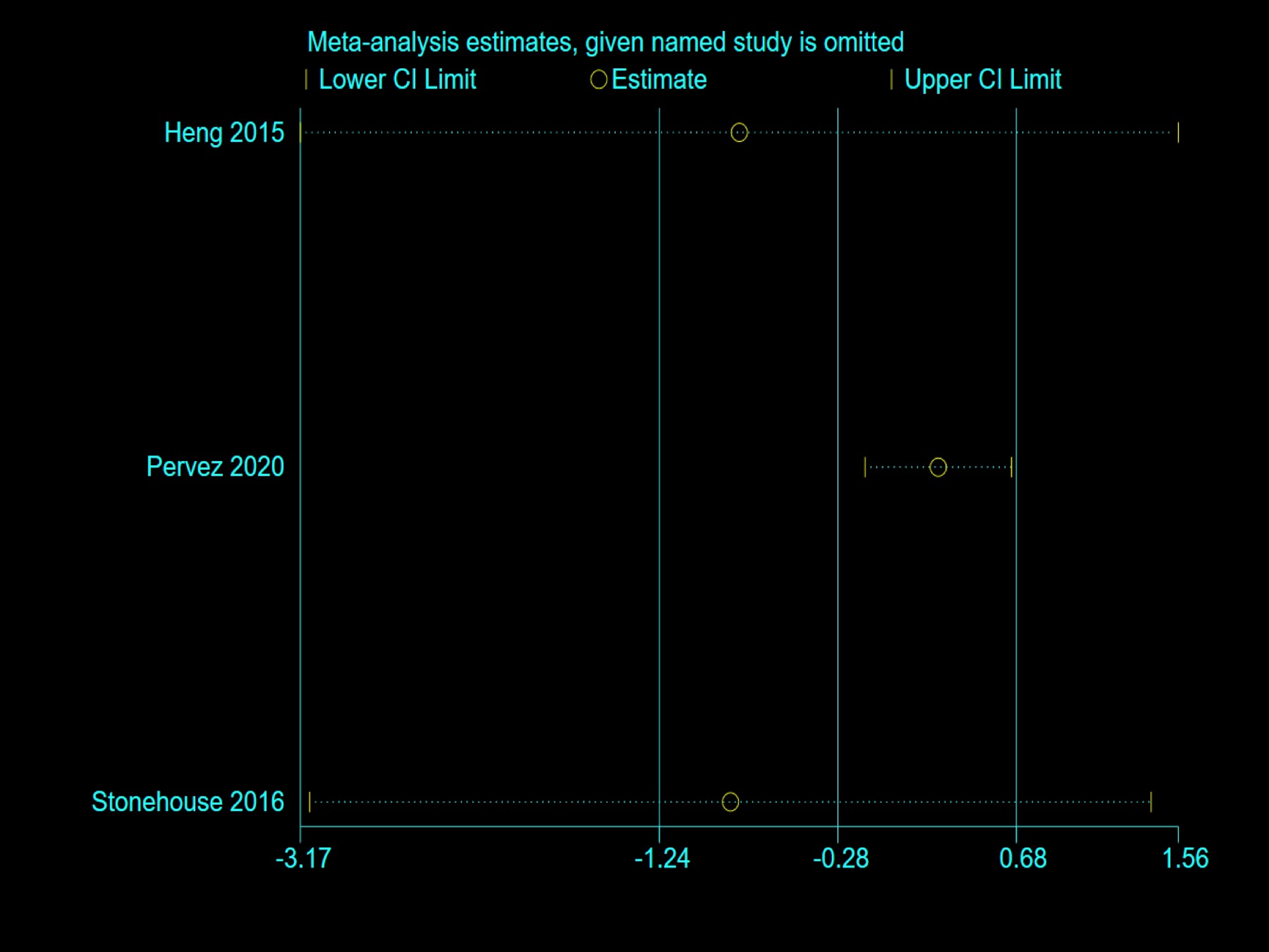

Supplement: S6 Fig — (TIF) [file pone.0255205.s007.tif]

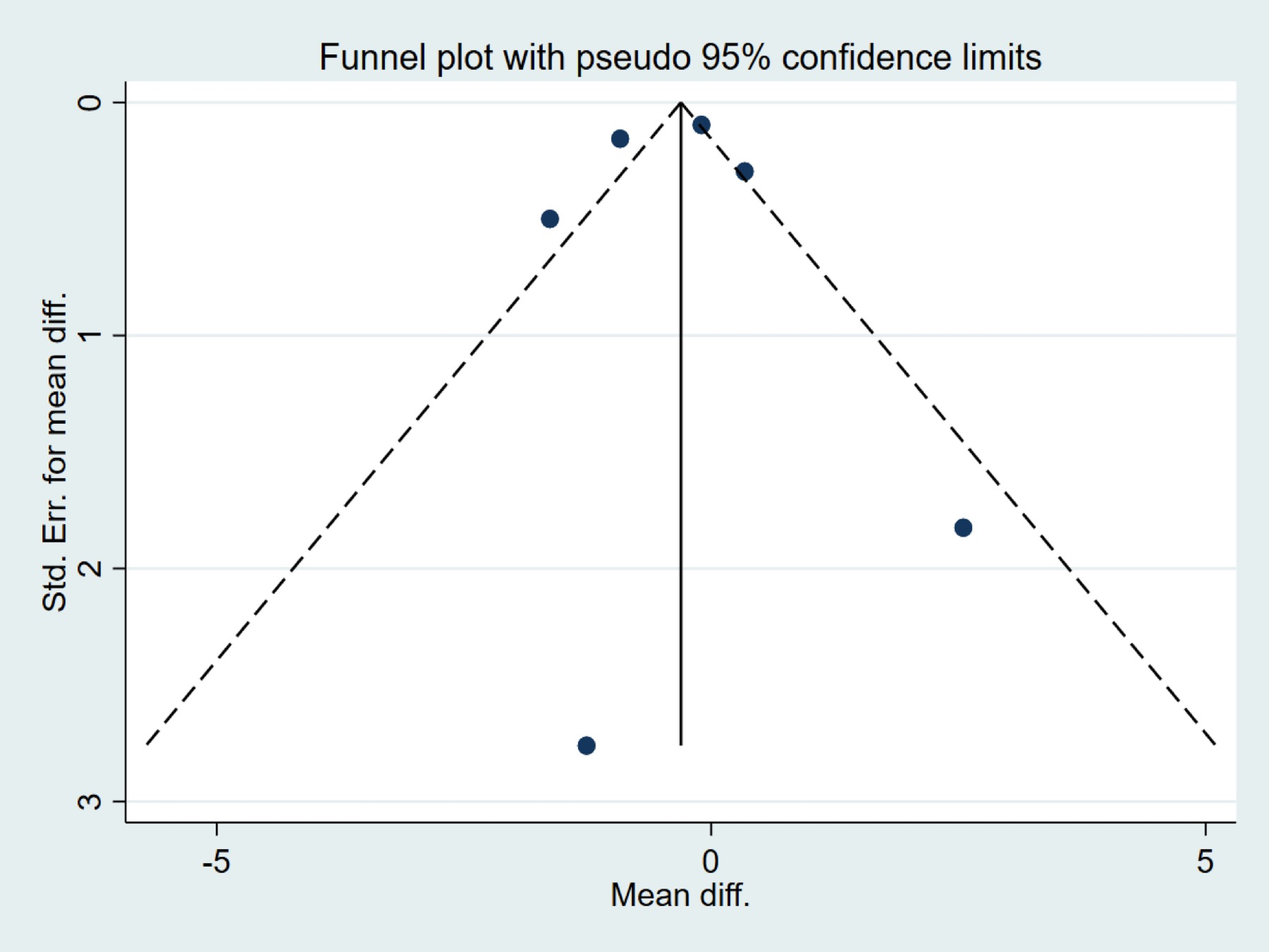

Supplement: S7 Fig — (TIF) [file pone.0255205.s008.tif]

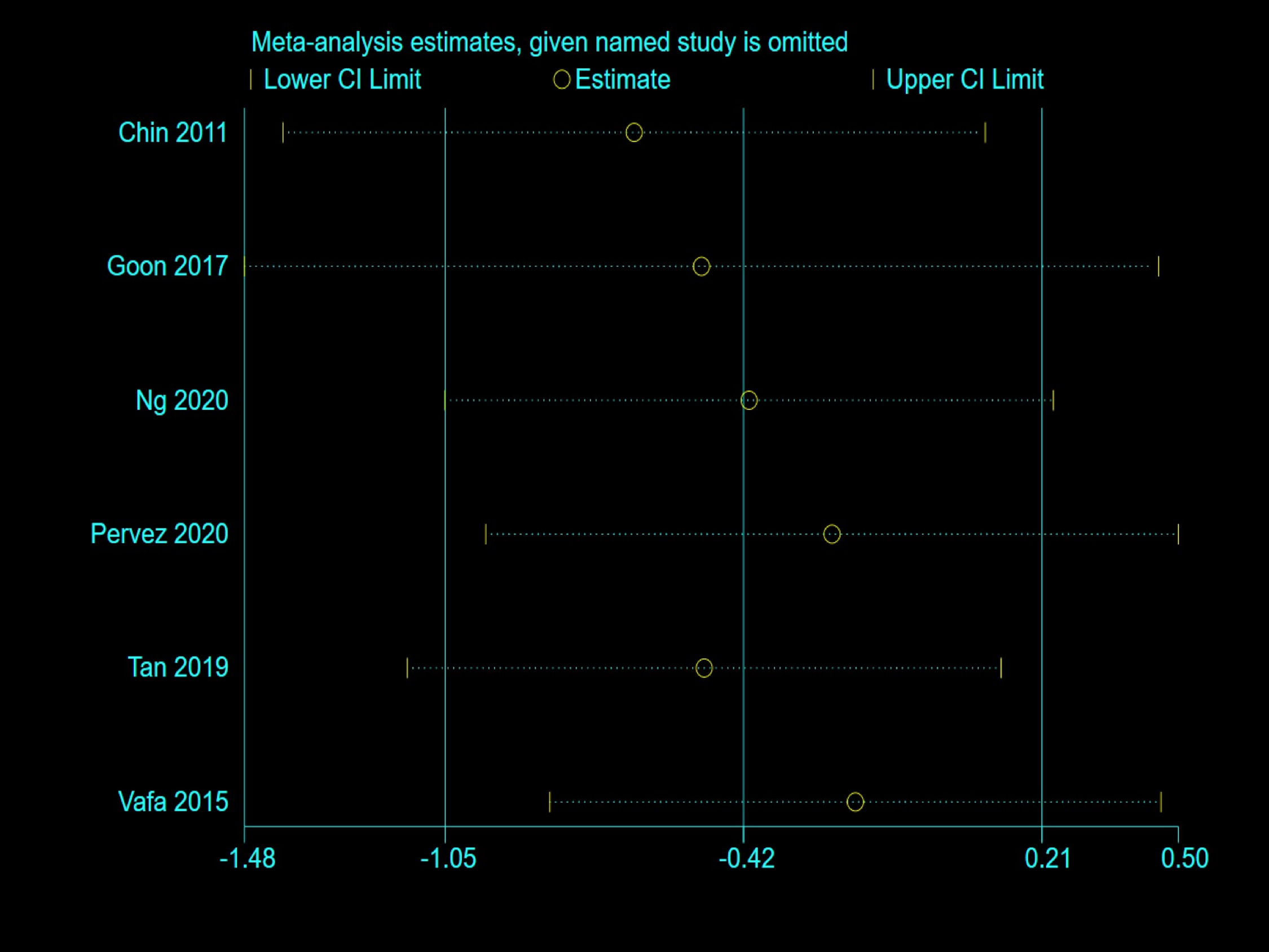

Supplement: S8 Fig — (TIF) [file pone.0255205.s009.tif]
