# Supplementary material for: Effects of tocotrienols supplementation on markers of inflammation and oxidative stress: A systematic review and meta-analysis of randomized controlled trials
Source: PLoS One. 2021 Jul 23;16(7):e0255205. doi: 10.1371/journal.pone.0255205 (PMC8301652; doi:10.1371/journal.pone.0255205)
Supplement: S1 Table — (DOCX) [file pone.0255205.s010.docx]

Table S1 Full search strategy

| No. | Database | Search Terms and Results |
| --- | --- | --- |
| 1. | PubMed  (13 July 2020) | #1: (((("tocotrienols"[MeSH Terms]) OR (tocotrienol[Title/Abstract])) OR (tocotrienol-rich vitamin E[Title/Abstract])) OR (tocotrienol-rich fraction[Title/Abstract])) OR (annatto-extracted tocotrienol[Title/Abstract]) **1385**  #2 (randomized controlled trial [pt] OR controlled clinical trial [pt] OR randomized [tiab] OR placebo [tiab] OR drug therapy [sh] OR randomly [tiab] OR trial [tiab] OR groups [tiab]) NOT (animals [mh] NOT humans [mh]) **4173488**  #3 #1 AND #2 **330** |
| 2. | Scopus (13 July 2020) | #1 INDEXTERMS (tocotrienol OR "tocotrienol-rich fraction" OR "tocotrienol-rich vitamin E" OR "annatto tocotrienol")OR TITLE-ABS-KEY(tocotrienol OR "tocotrienol-rich fraction" OR "tocotrienol-rich vitamin E" OR "annatto tocotrienol") **3151**  #2 (INDEXTERMS ("clinical trials" OR "clinical trials as a topic" OR "randomized controlled trial" OR "Randomized Controlled Trials as Topic" OR "controlled clinical trial" OR "Controlled Clinical Trials" OR "random allocation" OR "Double-Blind Method" OR "Single-Blind Method" OR "Cross-Over Studies" OR "Placebos" OR "multicenter study" OR "double blind procedure" OR "single blind procedure" OR "crossover procedure" OR "clinical trial" OR "controlled study" OR "randomization" OR "placebo")) OR (TITLE-ABS-KEY(("clinical trials" OR "clinical trials as a topic" OR "randomized controlled trial" OR "Randomized Controlled Trials as Topic" OR "controlled clinical trial" OR "Controlled Clinical Trials as Topic" OR "random allocation" OR "randomly allocated" OR "allocated randomly" OR "Double-Blind Method" OR "Single-Blind Method" OR "Cross-Over Studies" OR "Placebos" OR "cross-over trial" OR "single blind" OR "double blind" OR "factorial design" OR "factorial trial"))) OR (TITLE-ABS(clinical AND trial* OR trial* OR rct* OR random* OR blind*)) **7648625**  #3 #1 AND #2 **1118**  #4 KEY (nonhuman OR animal OR "animal experiment") **8709524**  #5 #3 NOT #4 **355** |
| 3. | Cochrane Library (13 July 2020) | #1 MeSH descriptor: [Tocotrienols] explode all trees **270**  #2 (tocotrienol OR “tocotrienol-rich fraction” OR “tocotrienol-rich vitamin E” OR “annatto tocotrienol”):ti.ab,kw **111**  #3 #1 OR #2 **330** |
