# Supplementary material for: Effects of tocotrienols supplementation on markers of inflammation and oxidative stress: A systematic review and meta-analysis of randomized controlled trials
Source: PLoS One. 2021 Jul 23;16(7):e0255205. doi: 10.1371/journal.pone.0255205 (PMC8301652; doi:10.1371/journal.pone.0255205)
Supplement: S2 Table — (DOCX) [file pone.0255205.s011.docx]

Table S2 Study selection based on inclusion criteria after reviewing full text

| No | First author, year | Journal | Tittle | Include? | Reason of Exclusion |
| --- | --- | --- | --- | --- | --- |
|  | Ablon, 2018 | Journal of Drugs in Dermatology | A six-month, randomized, double-blind, placebo-controlled study evaluating the safety and efficacy of a nutraceutical supplement for promoting hair growth in women with self-perceived thinning hair | No | No outcome of interest |
|  | Azman, 2018 | Antioxidants | Comparing palm oil, tocotrienol-rich fraction and α-tocopherol supplementation on the antioxidant levels of older adults | Yes |  |
|  | Baliarsingh, 2005 | Atherosclerosis | The therapeutic impacts of tocotrienols in type 2 diabetic patients with hyperlipidemia | No | No outcome of interest |
|  | Block 2004 | Journal of the American College of Nutrition | Plasma C-reactive protein concentrations in active and passive smokers: influence of antioxidant supplementation | No | Combination with other nutrients (Vitamin C) |
|  | Bloomer, 2007 | Journal of the International Society of Sports Nutrition | Prior exercise and antioxidant supplementation: effect on oxidative  stress and muscle injury | No | Acute study |
|  | Beoy, 2010 | Tropical Life Sciences Research | Effects of tocotrienol supplementation on hair growth in human volunteers | No | No outcome of interest |
|  | Byrne, 2000 | Free Radical Biology & Medicine | Studies of LDL oxidation following α-, γ-, or δ-tocotrienyl acetate supplementation of hypercholesterolemic humans | Yes |  |
|  | Casati, 2019 | Aging Clinical and Experimental Research | Vitamin E and Alzheimer’s disease: the mediating role of cellular aging | No | No control group |
|  | Catanzaro, 2016 | Hepatobiliary & Pancreatic Diseases International | Beneficial effect of refined red palm oil on lipid peroxidation and monocyte tissue factor in HCV-related liver disease: a randomized controlled study | No | Inappropriate control group (control group was healthy subjects but intervention group was patients with liver disease) |
|  | Che, 2017 | European Journal of Clinical Nutrition | Acute effects of a single dose of tocotrienols on insulinemic and inflammatory responses in metabolic syndrome subjects after a high-fat challenge | No | Acute study |
|  | Chen, 2006 | Journal of Sports Science and Medicine | Effects of palm vitamin E supplementation on exercise-induced oxidative stress and endurance performance in the heat | No | Acute study |
|  | Chin, 2008 | Nutrition | Reduction of DNA damage in older healthy adults by Tri E® Tocotrienol supplementation | No | No outcome of interest |
|  | Chin, 2011 | Nutrition & Metabolism | Tocotrienol rich fraction supplementation improved lipid profile and oxidative status in healthy older adults: A randomized controlled study | Yes |  |
|  | Choudhury, 1997 | Annals of Nutrition & Metabolism | Comparison of plasma lipids and vitamin E in young and middle-aged subjects on potato crisps fried in palmolein and highly oleic sunflower oil | No | Non-tocotrienols |
|  | Cicero, 2011 | Mediterranean Journal of Nutrition and Metabolism | Evaluation of the short term efficacy and tolerability of a combined nutraceutical with lipid-lowering properties: a randomized clinical trial | No | No outcome of interest |
|  | Cicero, 2015 | Nutrition Journal | Short-term effects of a combined nutraceutical of insulin-sensitivity, lipid level and indexes of liver steatosis: a double-blind, randomized, cross-over clinical trial | No | No outcome of interest |
|  | Daud, 2013 | Vascular Health and Risk Management | Vitamin E tocotrienol supplementation improves lipid profiles in chronic hemodialysis patients | Yes |  |
|  | Gan, 2017 | Scientific Reports | Effect of palm-based tocotrienols and tocopherol mixture supplementation on platelet aggregation in subjects with metabolic syndrome: a randomized controlled trial | Yes |  |
|  | Ghani, 2019 | Clinics | Comparing the effects of vitamin E tocotrienol-rich fraction supplementation and α-tocopherol supplementation on gene expression in healthy older adults | No | No outcome of interest |
|  | Goon, 2017 | Clinical Nutrition ESPEN | Comparing palm oil tocotrienol rich fraction with α-tocopherol supplementation on oxidative stress in healthy older adults | Yes |  |
|  | Gopalan, 2014 | Stroke | Clinical investigation of the protective effects of palm vitamin E tocotrienols on brain white matter | Yes |  |
|  | Grau, 2015 | J Rev Argent Dermatosifilol | Effectiveness of a dietary supplement with serenoa serrulata and tocotrienol-tocopherol against female androgenetic alopecia and telogen effluvium. Report of a pilot study | No | Non-English literature |
|  | Haghighat, 2013 | International Journal of Preventive Medicine | The effects of tocotrienols added to canola oil on microalbuminuria,  inflammation, and nitrosative stress in patients with type 2 diabetes:  a randomized, double-blind, placebo-controlled trial | Yes |  |
|  | Hamilton, 2019 | Journal of Pain Research | Pain reduction and improved vascular health associated with daily consumption of an anti-inflammatory dietary supplement blend | No | No control group |
|  | Heng, 2013 | European Journal of Nutrition | Supplementation with tocotrienol-rich fraction alters the plasma levels of Apolipoprotein A-I precursor, Apolipoprotein E precursor, and C-reactive protein precursor from young and old individuals | No | No outcome of interest |
|  | Heng, 2015 | Malaysian Journal of Nutrition | Potential of mixed tocotrienol supplementation to reduce cholesterol and cytokines level in adults with metabolic syndrome | Yes |  |
|  | Hor, 2018 | JAMA Neurology | Efficacy of oral mixed tocotrienols in diabetic peripheral neuropathy: A randomized clinical trial | No | No outcome of interest |
|  | Jubri, 2013 | Nutrition Journal | Perturbation of cellular immune functions in cigarette smokers and protection by palm oil vitamin E supplementation | No | No outcome of interest |
|  | Kooyenga, 1997 | Asia Pacific Journal of Clinical Nutrition | Palm oil antioxidant effects in patients with hyperlipidemia and carotid stenosis-2 year experience | Yes |  |
|  | Lietz, 2006 | Journal of Nutrition | Xanthophyll and hydrocarbon carotenoid patterns differ in plasma and breast milk of women supplemented with red palm oil during  pregnancy and lactation | No | No outcome of interest |
|  | Lin, 2016 | Journal of Oil Palm Research | Safety assessment of tocotrienol supplementation subjects with metabolic syndrome a randomized control trial | No | No outcome of interest |
|  | Liu, 2018 | Journal of Cachexia, Sarcopenia and Muscle | Building strength, endurance, and mobility using an astaxanthin formulation with functional training in elderly | No | No outcome of interest |
|  | Magosso, 2013 | Nutrition Journal | Tocotrienols for normalisation of hepatic echogenic response in nonalcoholic fatty liver: a randomised placebo-controlled clinical trial | Yes |  |
|  | Mahalingam, 2011 | European Journal of Clinical Nutrition | Effects of supplementation with tocotrienol-rich fraction on immune response to tetanus toxoid immunization in normal healthy volunteers | No | No outcome of interest |
|  | Mahdy, 2013 | Acta Medica | Does palm oil vitamin E reduce the risk of pregnancy induced hypertension | No | No outcome of interest |
|  | Maki, 2015 | Prostaglandins, Leukotrienes & Essential Fatty Acids | Safety and lipid-altering efficacy of a new omega-3 fatty acid and antioxidant containing medical food in men and women with elevated triacylglycerols | No | Combination with other nutrients (omega-3 fatty acids) |
|  | Mensink, 1999 | American Journal of Clinical Nutrition | A vitamin E concentrate rich in tocotrienols had no effect on serum lipids, lipoproteins, or platelet function in men with mildly elevated serum lipid concentrations | No | No outcome of interest |
|  | Muid, 2017 | International Food Research Journal | Tocotrienol rich fraction supplement reduces oxidative stress in non familial hypercholesterolaemia: beyond the lipid lowering capability | No | Inappropriate control group (control group received statin) |
|  | Mustad, 2002 | American Journal of Clinical Nutrition | Supplementation with 3 compositionally different tocotrienol supplements does not improve cardiovascular disease risk factors in men and women with hypercholesterolemia | No | No outcome of interest |
|  | Nazaimoon, 1996 | Nutrition Research | Effects of palm olein tocopherol and tocotrienol on lipid peroxidation, lipid profiles and glycemic control in non-insulin diabetes mellitus patients | Yes |  |
|  | Nesaretnam, 2010 | Breast Cancer Research | Effectiveness of tocotrienol-rich fraction combined with tamoxifen in the management of women with early breast cancer: a pilot clinical trial | No | No outcome of interest |
|  | Ng, 2020 | Nutrients | The effects of tocotrienol-rich vitamin E (Tocovid) on diabetic neuropathy: a phase ii randomized controlled trial | Yes |  |
|  | Orozco, 2010 | Journal of Nutrition | Antioxidant-rich oral supplements attenuate the effects of oral iron on in situ oxidation susceptibility of human feces | No | No outcome of interest |
|  | Orozco, 2012 | Food and Nutrition Bulletin | Response of urinary biomarkers of systemic oxidation to oral iron supplementation in healthy men | No | No outcome of interest |
|  | Osman, 2016 | Biomedical Research and Therapy | Effects of adding tocotrienol-tocopherol mixed fraction and vitamin C on inflammatory status in hypercholesterolaemic patients in the low coronary risk category | No | Statistical analyses included subjects receiving combination with vitamin C |
|  | Patel, 2012 | Journal of Nutrition | Oral tocotrienols are transported to human tissues and delay the progression of the model for end-stage liver disease score in patients | No | No outcome of interest |
|  | Pervez, 2018 | Turkish Journal of Gastroenterology | Effects of delta-tocotrienol supplementation on liver enzymes, inflammation, oxidative stress and hepatic steatosis in patients with nonalcoholic fatty liver disease | No | Duplication publication (Pervez 2020) |
|  | Pervez, 2020 | Complementary Therapies in Medicine | Delta-tocotrienol supplementation improves biochemical markers of hepatocellular injury and steatosis in patients with nonalcoholic fatty liver disease: A randomized, placebo-controlled trial | Yes |  |
|  | Qureshi, 1991 | American Journal of Clinical Nutrition | Lowering of serum cholesterol in hypercholesterlemic humans by tocotrienols (palmvitee) | No | No outcome of interest |
|  | Qureshi, 1995 | Lipids | Response of hypercholesterolemic subjects to administration of tocotrienols | No | No outcome of interest |
|  | Qureshi, 1997 | Nutritional Biochemistry | Novel tocotrienols of rice bran modulate cardiovascular disease risk parameters of hypercholesterolemic humans | No | No outcome of interest |
|  | Qureshi, 2001 | Journal of Nutritional Biochemistry | Synergistic effect of tocotrienol-rich fraction (TRF_25_) of rice bran and lovastatin on lipid parameters in hypercholesterolemic humans | No | No outcome of interest |
|  | Qureshi, 2002 | Atherosclerosis | Dose-dependent suppression of serum cholesterol by tocotrienol-rich fraction (TRF_25_) of rice bran in hypercholesterolemic humans | No | No outcome of interest |
|  | Qureshi, 2012 | Journal of Clinical & Experimental Cardiology | Suppression of nitric oxide production and cardiovascular risk factors in healthy seniors and hypercholesterolemic subjects by a combination of polyphenols and vitamins | No | No control group |
|  | Qureshi, 2013 | Journal of Clinical & Experimental Cardiology | Butritional supplement-5 with a combination of proteasome inhibitors  (Resveratrol, Quercetin, δ-tocotrienol) modulate age-associated  biomarkers and cardiovascular lipid parameters in human subjects | No | Combination with other nutrients (resveratrol and quercetin) |
|  | Radhakrishnan, 2009 | British Journal of Nutrition | Daily supplementation of tocotrienol-rich fraction or a-tocopherol did not induce immunomodulatory changes in healthy human volunteers | No | No outcome of interest |
|  | Rasool, 2006 | Journal of Nutritional Science and Vitaminology | Dose dependent elevation of plasma tocotrienol levels and its effect on arterial compliance, plasma total antioxidant status, and lipid profile in healthy humans supplemented with tocotrienol rich vitamin E | Yes |  |
|  | Rasool, 2008 | Archives of Pharmacal Research | Arterial compliance and vitamin E blood levels with a self emulsifying  preparation of tocotrienol rich vitamin E | No | No outcome of interest |
|  | Roza, 2007 | Alternative Therapies | Effect of citrus flavonoids and tocotrienols on serum cholesterol levels in hypercholesterolemic subjects | No | No outcome of interest |
|  | Schuchardt, 2015 | European Journal of Clinical Nutrition | A combination of palm oil tocotrienols and citrus peel polymethoxylated flavones does not influence elevated LDL qcholesterol and high-sensitivity C-reactive protein levels | No | Combination with other nutrients (flavones) |
|  | Shen, 2018 | BMC Complementary and Alternative Medicine | A 12-week evaluation of annatto tocotrienol supplementation for postmenopausal women: safety, quality of life, body composition, physical activity, and nutrient intake | No | No outcome of interest |
|  | Shen, 2018 | Osteoporosis International | Tocotrienol supplementation suppressed bone resorption and oxidative stress in postmeonopausal osteopenic women: a 12-week randomized double-blind placebo-controlled trial | No | No outcome of interest |
|  | Springett, 2015 | EBioMedicine | A phase I safety, pharmacokinetic, and pharmacodynamic presurgical trial of vitamin E δ-tocotrienol in patients with pancreatic ductal neoplasia | No | Not a RCT |
|  | Stonehouse, 2016 | Atherosclerosis | Short term effects of palm-tocotrienol and palm-carotenes on vascular function and cardiovascular disease risk: A randomised controlled trial | Yes |  |
|  | Tan, 2018 | Nutrients | Tocotrienol-rich vitamin e from palm oil (Tocovid) and its effects in diabetes and diabetic nephropathy: a pilot phase II clinical trial | Yes |  |
|  | Tan, 2019 | Therapeutic Advances in Endocrinology and Metabolism | Tocotrienol-rich vitamin E improves diabetic nephropathy and persists 6–9 months after washout: a phase IIa randomized controlled trial | Yes |  |
|  | Thomsen, 2019 | Pharmacological Research | Delta tocotrienol in recurrent ovarian cancer. A phase II trial | No | Not a RCT |
|  | Tomeo, 1995 | Lipids | Antioxidant effects of tocotrienols in patients with hyperlipidemia and carotid stenosis | No | Duplicate publication (Kooyenga 1997) |
|  | Vafa, 2015 | Journal of Research in Medical Sciences | Effect of tocotrienols enriched canola oil on glycemic control and oxidative status in patients with type 2 diabetes mellitus: A randomized double-blind placebo-controlled clinical trial | Yes |  |
|  | Wahlqvist, 1992 | Nutrition Research | Differential serum responses of tocopherols and tocotrienols during vitamin supplementation in hypercholesterolaemic individuals without change in coronary risk factors | No | No outcome of interest |
|  | Zahara, 2010 | Clinical Therapeutics | Plasma vitamin C and tocotrienols changes in response to dietary supplementation among young male adults | No | No outcome of interest |
|  | Zaiden, 2010 | Journal of Atherosclerosis and Thrombosis | Gamma delta tocotrienols reduce hepatic triglyceride synthesis and VLDL secretion | No | No outcome of interest |
|  | Zesiewicz, 2018 | Neurodegenerative Disease Management | Double-blind, randomized and controlled trial of EPI-743 in Friedreich’s ataxia | No | Non-tocotrienols |
