# Supplementary material for: Effects of tocotrienols supplementation on markers of inflammation and oxidative stress: A systematic review and meta-analysis of randomized controlled trials
Source: PLoS One. 2021 Jul 23;16(7):e0255205. doi: 10.1371/journal.pone.0255205 (PMC8301652; doi:10.1371/journal.pone.0255205)
Supplement: S3 Table — (DOCX) [file pone.0255205.s012.docx]

Table S3 Additional information on the tocotrienols supplements used in all studies

| No. | Author, year | Brand | T3 Isomers (mg/d) | Manufacturer |
| --- | --- | --- | --- | --- |
|  | Azman, 2018; Goon 2017 | N/A | α-T3 (70.4 mg), β-T3 (4.8 mg), γ-T3 (57.6 mg), δ-T3 (33.6 mg) | Sime Darby Food and Beverages Marketing Sdn. Bhd, Malaysia |
|  | Bryne, 2000 | Tocomin 50 | Group 1: α-tocotrienyl acetate (148 mg)  Group 2: γ-tocotrienyl acetate (140 mg)  Group 3: δ-tocotrienyl acetate (119 mg) and γ-tocotrienyl acetate (17 mg) | Carotech, Malaysia |
|  | Chin, 2011 | Tri E® Tocotrienol | α-T3 (70.4 mg), β-T3 (4.8 mg), γ-T3 (57.6 mg), δ-T3 (33.6 mg) | Sime Darby Bioganic Sdn. Bhd., Malaysia |
|  | Daud, 2013 | N/A | α-T3 (60.4 mg), β-T3 (10.6 mg), γ-T3 (83.3 mg), δ-T3 (25.7 mg) | Carotino Sdn. Bhd., Malaysia |
|  | Gan, 2017, Gopalan, 2014, Heng, 2015, Magosso, 2013, Ng 2020, Tan 2018, Tan 2019 | Tocovid^TM^ SupraBio^TM^ | α-T3 (123.0 mg), γ-T3 (225.6 mg), δ-T3 (51.4 mg) | Hovid Bhd., Malaysia |
|  | Nazaimoon, 1996; Kooyenga, 1997 | Palmvitee | N/A | Palm Oil Research Institute Malaysia |
|  | Pervez, 2020 | N/A | γ-T3 (540 mg), δ-T3 (60 mg) | American River Nutrition, USA |
|  | Rasool, 2006 | N/A | Group 1: α-T3 (27.7 mg), γ-T3 (19.7 mg), δ-T3 (12.0 mg)  Group 2: α-T3 (55.4 mg), γ-T3 (39.4 mg), δ-T3 (24.0 mg)  Group 3: α-T3 (110.7 mg), γ-T3 (78.7 mg), δ-T3 (48.0 mg) | Golden Hope Plantation Bhd. (Sime Darby Bioganic Sdn. Bhd.), Malaysia |
|  | Stonehouse, 2016 | TRF-80 | α-T3 (135.2 mg), β-T3 (19.4 mg), γ-T3 (195.4 mg), δ-T3 (70 mg) | Carotino Sdn. Bhd., Malaysia |
|  | Haghighat, 2013; Vafa, 2015 | Tocomas | α-T3 (69.3 mg), γ-T3 (87.2 mg) | Musim Mas, Malaysia |

Abbreviation: N/A, not available; T3, tocotrienols.
